# Inflammatory and tissue injury marker dynamics in pediatric acute respiratory distress syndrome

**DOI:** 10.1172/JCI177896

**Published:** 2024-04-04

**Authors:** Nadir Yehya, Thomas J. Booth, Gnana D. Ardhanari, Jill M. Thompson, L.K. Metthew Lam, Jacob E. Till, Mark V. Mai, Garrett Keim, Daniel J. McKeone, E. Scott Halstead, Patrick Lahni, Brian M. Varisco, Wanding Zhou, Erica L. Carpenter, Jason D. Christie, Nilam S. Mangalmurti

**Affiliations:** 1 Division of Pediatric Critical Care, Department of Anesthesiology and Critical Care Medicine, Children’s Hospital of Philadelphia and; 2 Leonard Davis Institute of Health Economics, University of Pennsylvania, Philadelphia, Pennsylvania, USA.; 3 Division of Pediatric Cardiac Critical Care Medicine, Children’s Heart Institute, Memorial Hermann Hospital, University of Texas Health McGovern Medical School, Houston, Texas, USA.; 4 Division of Pulmonary, Allergy, and Critical Care, Department of Medicine, Department of Medicine and; 5 Division of Hematology-Oncology, Department of Medicine, Abramson Cancer Center, Perelman School of Medicine, University of Pennsylvania, Philadelphia, Pennsylvania, USA.; 6 Division of Pediatric Critical Care Medicine, Department of Pediatrics, Children’s Healthcare of Atlanta and Emory University, Atlanta, Georgia, USA.; 7 Division of Pediatric Hematology and Oncology, Department of Pediatrics and; 8 Division of Pediatric Critical Care Medicine, Department of Pediatrics, Pennsylvania State University College of Medicine, Hershey, Pennsylvania, USA.; 9 Division of Critical Care Medicine, Department of Pediatrics, Cincinnati Children’s Hospital Medical Center and University of Cincinnati College of Medicine, Cincinnati, Ohio, USA.; 10 Section of Critical Care, Department of Pediatrics, University of Arkansas for Medical Sciences and Arkansas Children’s Research Institute, Little Rock, Arkansas, USA.; 11 Center for Computational and Genomic Medicine, Children’s Hospital of Philadelphia, Philadelphia, Pennsylvania, USA.; 12 Center for Translational Lung Biology and; 13 Center for Clinical Epidemiology and Biostatics, University of Pennsylvania, Philadelphia, Pennsylvania, USA.

**Keywords:** Inflammation, Pulmonology, Endothelial cells, Innate immunity, Proteomics

## Abstract

**BACKGROUND:**

The molecular signature of pediatric acute respiratory distress syndrome (ARDS) is poorly described, and the degree to which hyperinflammation or specific tissue injury contributes to outcomes is unknown. Therefore, we profiled inflammation and tissue injury dynamics over the first 7 days of ARDS, and associated specific biomarkers with mortality, persistent ARDS, and persistent multiple organ dysfunction syndrome (MODS).

**METHODS:**

In a single-center prospective cohort of intubated pediatric patients with ARDS, we collected plasma on days 0, 3, and 7. Nineteen biomarkers reflecting inflammation, tissue injury, and damage-associated molecular patterns (DAMPs) were measured. We assessed the relationship between biomarkers and trajectories with mortality, persistent ARDS, or persistent MODS using multivariable mixed effect models.

**RESULTS:**

In 279 patients (64 [23%] nonsurvivors), hyperinflammatory cytokines, tissue injury markers, and DAMPs were higher in nonsurvivors. Survivors and nonsurvivors showed different biomarker trajectories. IL-1α, soluble tumor necrosis factor receptor 1, angiopoietin 2 (ANG2), and surfactant protein D increased in nonsurvivors, while DAMPs remained persistently elevated. ANG2 and procollagen type III N-terminal peptide were associated with persistent ARDS, whereas multiple cytokines, tissue injury markers, and DAMPs were associated with persistent MODS. Corticosteroid use did not impact the association of biomarker levels or trajectory with mortality.

**CONCLUSIONS:**

Pediatric ARDS survivors and nonsurvivors had distinct biomarker trajectories, with cytokines, endothelial and alveolar epithelial injury, and DAMPs elevated in nonsurvivors. Mortality markers overlapped with markers associated with persistent MODS, rather than persistent ARDS.

**FUNDING:**

NIH (K23HL-136688, R01-HL148054).

## Abstract 2

The longitudinal biomarker profile of pediatric acute respiratory distress syndrome suggests that organ failures and hyperinflammation are the primary drivers of mortality.

## Introduction

Acute respiratory distress syndrome (ARDS) is a heterogeneous condition of proteinaceous pulmonary edema causing acute life-threatening hypoxemia. Primarily described for adults (1, 2), pediatric ARDS has a distinct epidemiology (3, 4). Moreover, it is unclear whether the molecular mechanisms underlying the development and progression of pediatric ARDS are comparable to what is known in adults. In adult ARDS, upstream damage-associated molecular patterns (DAMPs) (5, 6), the initial hyperinflammatory response (7, 8), alveolar epithelial damage (9, 10), and endotheliopathy (11–13) have all been implicated to varying degrees in ARDS pathophysiology. However, the molecular signature of pediatric ARDS is less understood, and the degree to which the hyperinflammatory response or injuries to specific tissues contributes to outcomes in children is unknown.

In both adults and pediatric ARDS, plasma biomarkers have been proposed as a method to reduce heterogeneity, with consistent demonstration of hypo- and hyperinflammatory subphenotypes defined by (primarily) innate immunity cytokines (interleukin 6 [IL-6], IL-8, soluble tumor necrosis factor receptor 1 [sTNFR1]) and metrics of shock severity (vasopressor use, bicarbonate) (14–22). These subphenotypes have clinical utility for prognostic, and potentially for predictive, enrichment strategies in future trials. The relative importance of innate immunity and shock biomarkers in defining these subphenotypes, relative to lung epithelial markers, suggests that a focus on systemic hyperinflammatory biomarkers is warranted for both adult and pediatric ARDS. Few studies, however, have assessed the longitudinal trajectory of plasma biomarkers and correlated them with the natural history of pediatric ARDS (23), and direct comparisons of DAMPs, cytokines, and tissue injury markers are lacking.

Understanding the longitudinal biochemical profile of pediatric ARDS would be informative for identifying targetable mechanisms in order to improve outcomes in future trials. Therefore, we investigated the evolution of pediatric ARDS by serially measuring inflammation and tissue injury markers over the first 7 days. We associated specific biomarkers representing DAMPs, cytokines, chemokines, plasma proteases, and tissue injury (Supplemental Table 1; supplemental material available online with this article; https://doi.org/10.1172/JCI177896DS1) with pediatric intensive care unit (PICU) mortality, persistent ARDS, and persistent multiple organ dysfunction syndrome (MODS). We hypothesized that specific biomarker trajectories would correlate with clinical trajectories in a manner that reflected progression of underlying pathophysiology.

## Results

### Description of the cohort.

**Figure 1:**
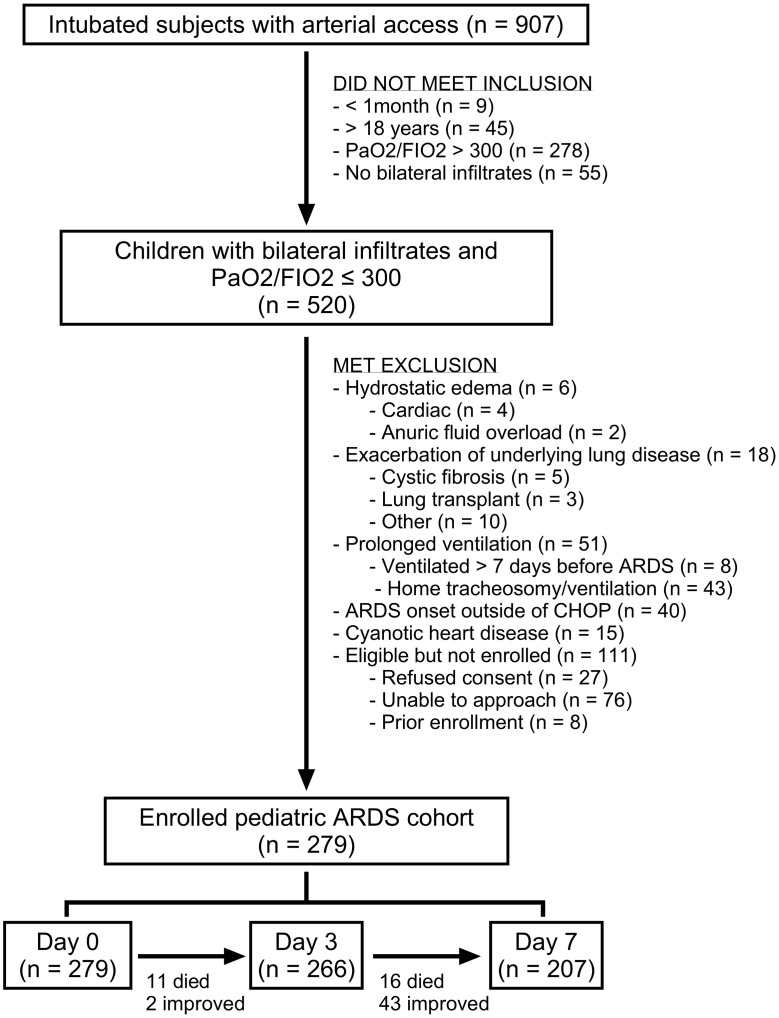
Study flowchart.

**Figure 2:**
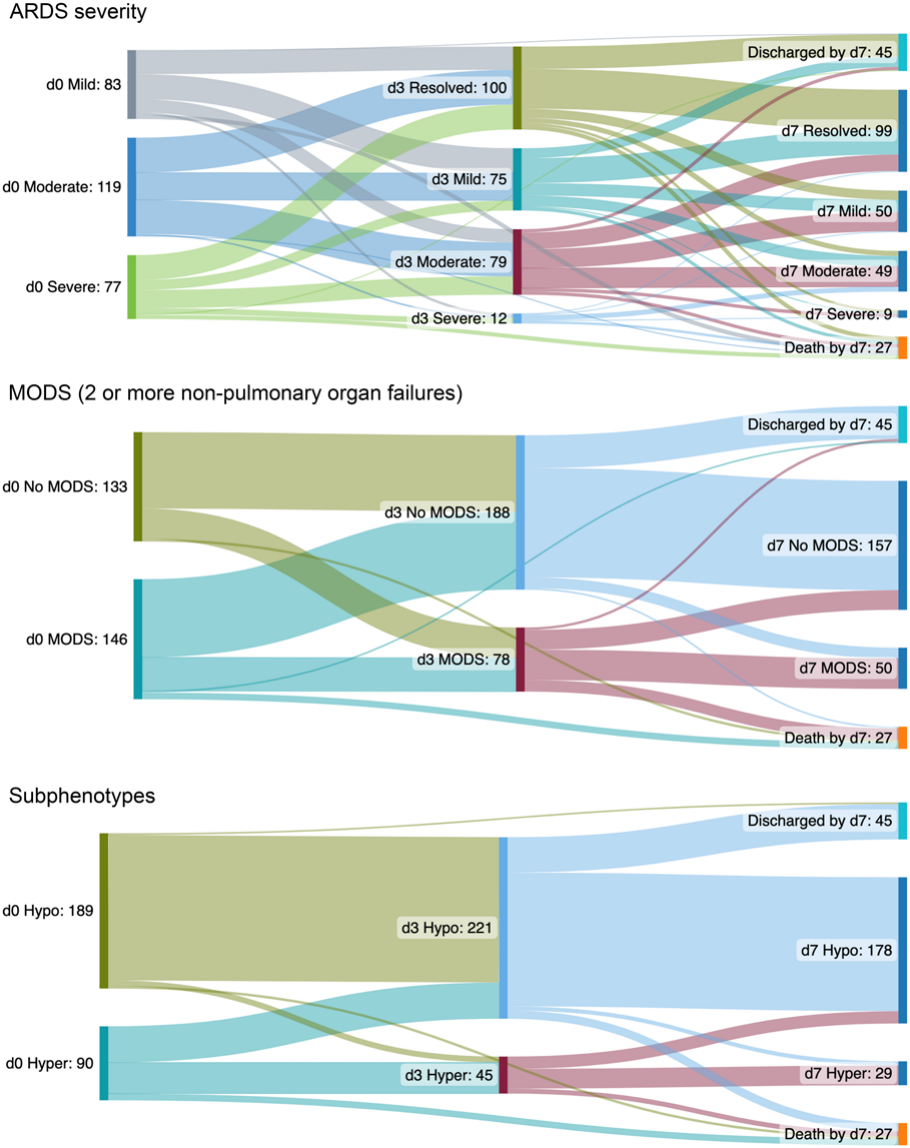
Clinical trajectories of ARDS severity (Berlin mild, moderate, severe), MODS, and hyper-/hypoinflammatory ARDS subphenotype (defined using a parsimonious algorithm of IL-6, IL-8, CCL3/MIP-1α, and ANG2) over the first 7 days. Top: Berlin ARDS trajectories are stratified according to whether patients have mild (gray), moderate (blue), or severe (green) on day 0; on day 3, patients are again restratified according to whether ARDS has resolved (olive), or is mild (aqua), moderate (red), or severe (blue) according to Berlin criteria. Middle: MODS trajectories are stratified according to whether patients have at least 2 nonpulmonary organ failures (aqua) or not (olive) on day 0; on day 3, patients are restratified according to whether they have at least 2 nonpulmonary organ failures (red) or not (blue). Bottom: Day 0 hypo- (olive) and hyperinflammatory (aqua) ARDS trajectories, and day 3 hypo- (blue) and hyperinflammatory (red) ARDS subphenotype are similarly labeled. By day 7, 45 patients had been discharged alive from the PICU, and 27 had died. Note that these 27 nonsurvivors within 7 days of ARDS onset represent a subset of the total (*n* = 64) who died in the PICU.

We enrolled 279 intubated and mechanically ventilated children meeting Berlin criteria for ARDS (2), with plasma collection on days 0, 3, and 7 after ARDS onset for biomarker measurements (Figure 1). The median age of the cohort was 6.8 years (IQR 2–13.5), 124 (44%) patients were female, and 64 (23%) patients were PICU nonsurvivors (Table 1). Nonsurvivors had greater illness severity, as defined by Pediatric Risk of Mortality (PRISM) III scores, more organ failures, higher vasopressor scores, were more likely to be immunocompromised, and were more likely to have nonpulmonary sepsis as an ARDS etiology (Table 1). There were 266 patients with plasma samples on day 3 and 207 on day 7 (Figure 1). Of the 13 patients unavailable for sampling by day 3, 11 were nonsurvivors; of the 72 patients unavailable by day 7, 27 were nonsurvivors (Figure 2). Prevalence of moderate/severe ARDS, MODS (at least 2 nonpulmonary organ failures), and hyperinflammatory ARDS was highest on day 0 (Figure 2). Trajectories of specific organ failures (cardiovascular, renal, hepatic, hematologic, neurologic) mirrored that of MODS, with most patients demonstrating resolution of organ failure over the first 7 days of ARDS (Supplemental Figure 1).

### Biomarker correlations.

**Figure 3:**
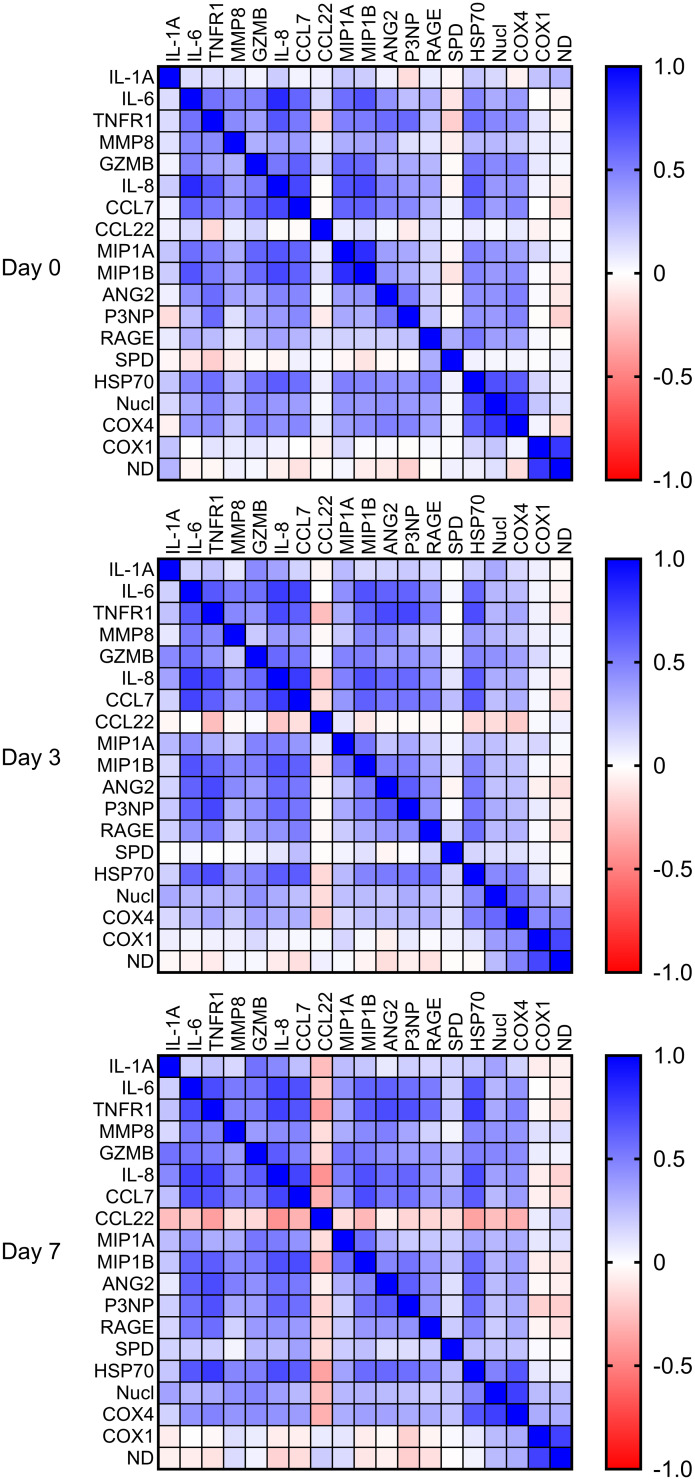
Correlation matrices on days 0, 3, and 7. Most biomarkers demonstrated modest (|*r*| between 0.3 and 0.7) correlation.

Of the measured biomarkers, most cytokines, proteases, chemokines, tissue injury markers, and DAMPs demonstrated modest correlation on days 0, 3, and 7 (Figure 3). C-C motif chemokine ligand 22 (CCL22), surfactant protein D (SPD), and mitochondrial DNAs (mtDNAs) (*COX1* and *ND1*) were least correlated with other biomarkers (|*r*| < 0.3), although SPD became more correlated with other biomarkers on day 7. DAMPs, tissue injury markers, and cytokines clustered together on days 0 and 3, with good separation between ARDS subphenotypes and eventual PICU nonsurvivors (Supplemental Figure 2).

### Biomarkers associated with PICU mortality.

**Figure 4:**
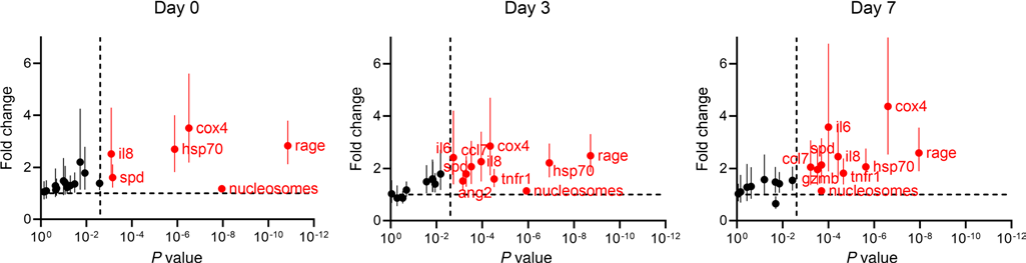
Differences in biomarkers of inflammation, tissue injury, and DAMPs between PICU survivors and nonsurvivors on days 0 (*n* = 279), 3 (*n* = 266), and 7 (*n* = 207) of ARDS. Values represent estimated levels (and 95% CIs) after biomarkers underwent log transformation and multivariable adjustment (age, ARDS etiology, immunocompromised status, initial PaO_2_/FIO_2_). The *y* axis shows differences in biomarker levels presented as a fold change; the *x* axis shows the *P* value. The dotted lines indicate a fold change = 1 (i.e., no difference; horizontal line) and the Bonferroni-corrected *P* value threshold (unadjusted *P* = 0.0025, Bonferroni-corrected *P* = 0.05; vertical line). This *P* value of the *t* statistic tests the hypothesis that the coefficient from the regression model differs from 0. Red dots depict biomarkers at unadjusted *P* < 0.0025; black dots represent those with unadjusted *P* > 0.0025.

On day 0, several DAMPs (nuclear DNA [nDNA] *COX4*, nucleosomes, heat shock protein 70 [HSP70]), tissue injury markers (SPD, soluble receptor for advanced glycation end-products [sRAGE]), and IL-8 were elevated in nonsurvivors in multivariable analysis (Figure 4). Notably, SPD and sRAGE are prominently expressed in alveolar epithelial cells. By day 3, these same markers remained elevated in nonsurvivors, as were the cytokines IL-6 and sTNFR1, the chemokine CCL7, and the endothelial damage marker angiopoietin 2 (ANG2). By day 7, these day 3 markers remained elevated in nonsurvivors, as was the protease granzyme B.

**Figure 5:**
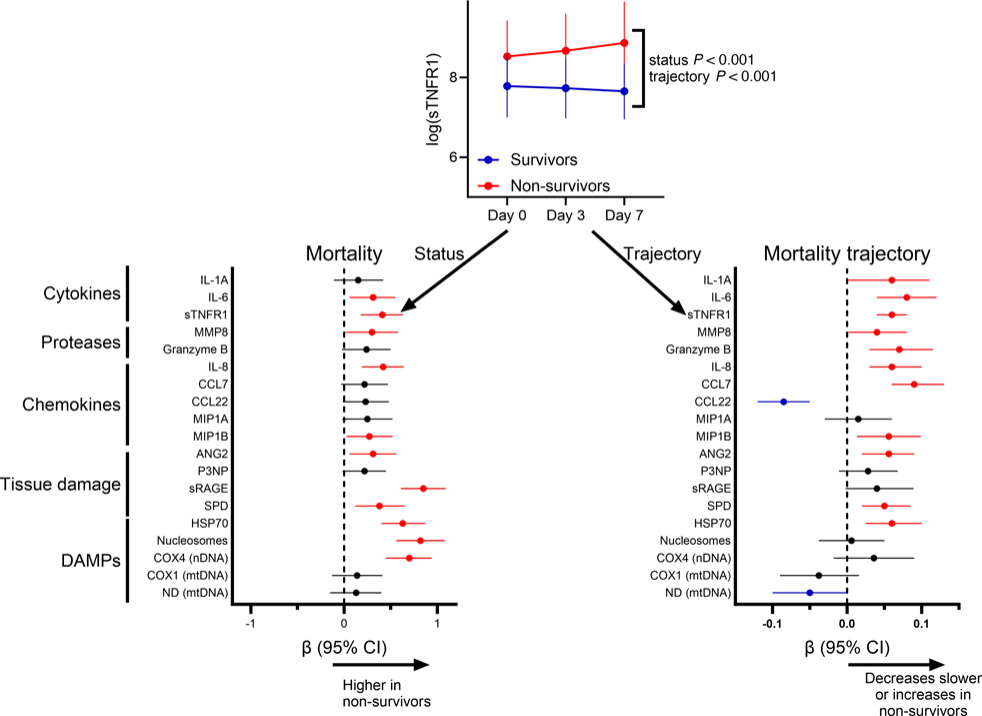
Association between biomarker levels and trajectory over the first 7 days of ARDS with PICU mortality. The β coefficients (and 95% CIs) are plotted for the association between the overall biomarker level in the first 7 days of ARDS (“status” in sTNFR1 example) and the trajectory (“trajectory” or status × time interaction term) with PICU mortality. In an effort to make meaningful comparisons between biomarkers, values are log transformed and standardized (set to mean = 0, SD = 1), and then adjusted for age, ARDS etiology, immunocompromised status, and initial PaO_2_/FIO_2_ in a multivariable mixed effects model. Red dots represent biomarkers with adjusted *P* < 0.05 with higher levels in nonsurvivors, blue dots represent biomarkers with adjusted *P* < 0.05 with lower levels in nonsurvivors, and black dots represent those with *P* > 0.05.

**Figure 6:**
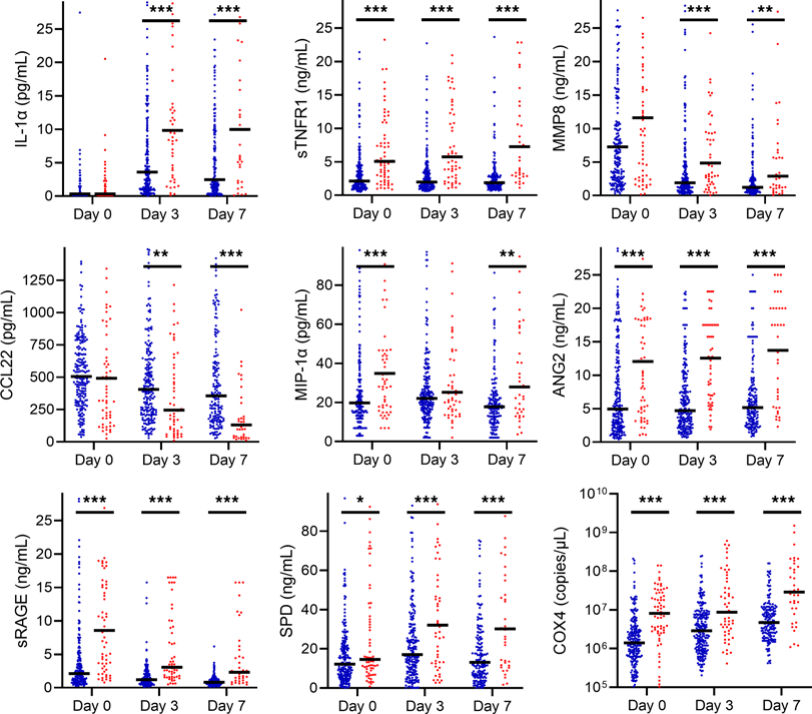
Unadjusted plasma biomarker levels between survivors (blue) and nonsurvivors (red) on days 0, 3, and 7 of ARDS. Black bars are median values. Unadjusted Wilcoxon’s rank sum tests compare survivors and nonsurvivors on days 0, 3, and 7 (*P < 0.05, **P < 0.01, ***P < 0.001). Select biomarkers are shown, with the remainder in Supplemental Figure 4.

PICU survivors and nonsurvivors demonstrated differing biomarker levels over the first 7 days of ARDS, as well as different trajectories, in adjusted mixed effects analyses (Figures 5 and 6 and Supplemental Figures 3 and 4). Multiple biomarkers of each class, particularly tissue injury markers and DAMPs (highest adjusted β coefficients), were elevated in nonsurvivors. Similarly, multiple cytokines, SPD, ANG2, and HSP70 increased or remained elevated in nonsurvivors. IL-6, IL-8, CCL7, and CCL22 decreased in all patients over the first 7 days, and CCL22 decreased faster in nonsurvivors. Results were similar when restricted to the 207 patients (thus excluding those who rapidly improved or died before day 7 and were unavailable for sampling) with samples collected at all 3 time points (Supplemental Figure 3).

### Biomarkers associated with persistent ARDS and with persistent MODS.

**Figure 7:**
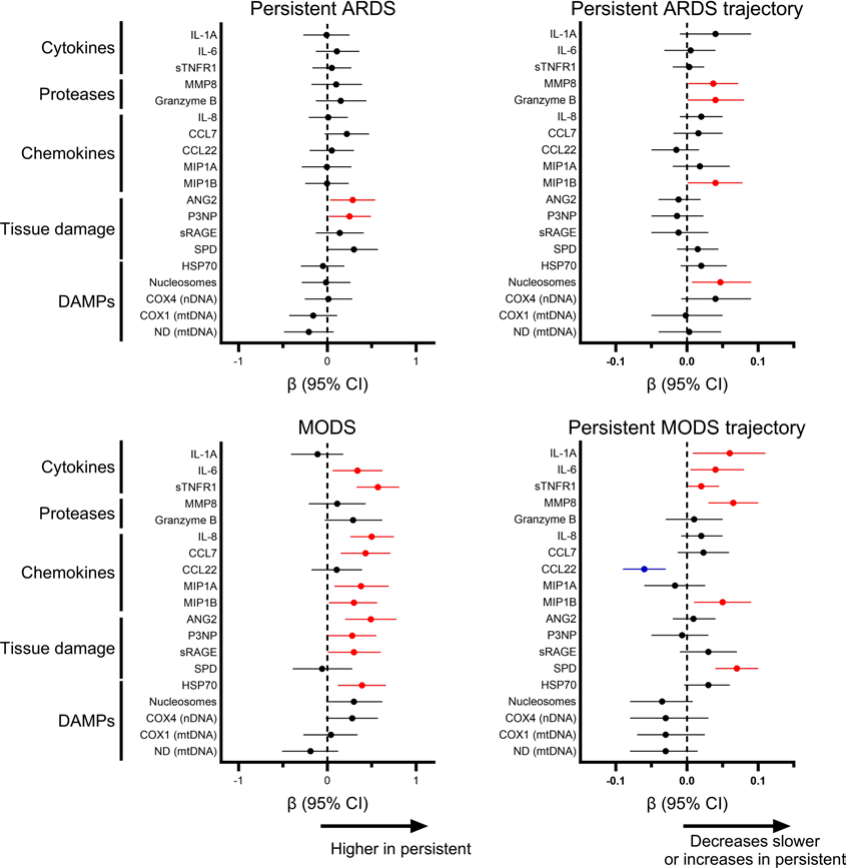
Association between biomarker levels and trajectory over the first 7 days of ARDS, with persistent ARDS (PaO_2_/FIO_2_ ≤ 200 on day 7) or persistent MODS (at least 2 nonpulmonary organ failures on day 7) restricted to patients who remained in the PICU until day 7 (*n* = 207). The β coefficients (and 95% CIs) are plotted for the association between the overall biomarker level in the first 7 days of ARDS and the trajectory with persistent ARDS and persistent MODS. Biomarker levels are log transformed and standardized (set to mean = 0, SD = 1), and then adjusted for age, ARDS etiology, immunocompromised status, and initial PaO_2_/FIO_2_ in a multivariable mixed effects model. Red dots represent biomarkers with adjusted *P* < 0.05 with higher levels in nonsurvivors, blue dots represent biomarkers with adjusted *P* < 0.05 with lower levels in nonsurvivors, and black dots represent those with *P* > 0.05.

Only 2 biomarkers, the tissue injury markers ANG2 and procollagen type III N-terminal peptide (P3NP), were higher over the first 7 days in patients with persistent ARDS (ratio of partial pressure of oxygen in arterial blood to the fraction of inspiratory oxygen concentration [PaO_2_/FIO_2_] ≤ 200 on day 7) (Figure 7). The proteases matrix metallopeptidase 8 (MMP8) and granzyme B, the chemokine macrophage inflammatory protein-1β (MIP-1β), and nucleosomes (DAMP) showed differential trajectory, either increasing or remaining elevated in patients with persistent ARDS.

By contrast, multiple cytokines, chemokines, and tissue injury markers were elevated in patients with persistent MODS (≥2 nonpulmonary organ failures on day 7) over the first 7 days (Figure 7). Patients with persistent MODS demonstrated increasing or persistently elevated levels of the cytokines IL-1α, IL-6, and sTNFR1, the protease MMP8, the chemokine MIP-1β, and the alveolar epithelial marker SPD. Overall, the biomarker profile of PICU nonsurvivors demonstrated greater overlap with that of persistent MODS, rather than with persistent ARDS.

### Biomarkers associated with direct/indirect ARDS.

As biomarkers differentiating different etiologies of ARDS have been described in adults (8), we also examined differences between direct (primarily pulmonary) and indirect (primarily nonpulmonary) ARDS. When examining differences in biomarker level and trajectory over the first 7 days of ARDS, most were higher in indirect ARDS; SPD was the sole biomarker higher in direct ARDS (Supplemental Figure 5). Notably, the alveolar epithelial markers SPD and sRAGE increased or remained persistently elevated over 7 days in indirect ARDS, whereas most chemokines, granzyme B, and DAMPs decreased more rapidly (from higher overall day 0 levels) in indirect ARDS.

### Biomarkers associated with baseline immune status.

As patients with a baseline immunocompromising condition may have a different biomarker profile, we analyzed whether adjusted biomarker levels and trajectories over the first 7 days of ARDS differed according to immunocompromised status. Immunocompromised patients (Supplemental Table 2) had more nonpulmonary organ failures at ARDS onset, were more likely to have nonpulmonary sepsis as an etiology, were more likely to receive corticosteroids, and had higher mortality. Immunocompromised patients had higher levels of nearly all biomarkers tested over the first 7 days of ARDS, with lower levels only of the protease MMP8 and the chemokine CCL22 (Supplemental Figure 6). Cytokines, chemokines, proteases, and tissue injury markers all increased or remained elevated longer in immunocompromised patients over the first 7 days of ARDS, whereas mtDNA levels decreased.

Immunocompetent patients demonstrated a biomarker profile in nonsurvivors over the first 7 days of ARDS that was similar to what was seen in the entire cohort (Supplemental Figure 7), with elevations in all biomarker classes, but with the highest levels (highest adjusted β coefficients) in tissue injury markers (ANG2 and sRAGE) and in DAMPs. By contrast, immunocompromised patients only showed elevations in tissue injury markers and DAMPs. Biomarker trajectories differed noticeably between immunocompetent and immunocompromised patients; immunocompetent patients only demonstrated decreasing CCL22 levels in nonsurvivors, whereas immunocompromised patients showed biomarker levels that were increasing (or were persistently elevated) for cytokines, chemokines, sRAGE (tissue injury), and DAMPs.

### Biomarkers associated with corticosteroid use.

As corticosteroid use could plausibly impact biomarker trajectories, we analyzed whether adjusted biomarker levels and trajectories over the first 7 days of ARDS differed according to exposure to systemic corticosteroids within the first 3 days of ARDS. We have previously shown that 90% of corticosteroid use occurs within the first 3 days of ARDS (24). Patients receiving corticosteroids were more likely to be immunocompromised, were more likely to have infectious pneumonia as an etiology, had worse lung mechanics at ARDS onset, and were more likely to receive other ancillary ARDS therapies (Supplemental Table 3). Over the first 7 days of ARDS, patients on corticosteroids had lower overall levels of cytokines and chemokines (Supplemental Figure 8), with falling levels of CCL22 and the endothelial damage marker ANG2, and rising levels of sTNFR1 and MIP-1β.

In both patients with and without corticosteroid use, nonsurvivors showed elevations primarily in tissue injury markers and DAMPs over the first 7 days of ARDS (Supplemental Figure 9). Patients with corticosteroid use had increasing trajectories of most biomarker classes, whereas patients without corticosteroid use only showed increases in sTNFR1 (cytokine) and CCL7 (chemokine). In patients both exposed and unexposed to corticosteroids, the chemokine CCL22 decreased more rapidly in nonsurvivors. Overall, biomarker levels and trajectories over the first 7 days of ARDS demonstrated similar associations with mortality for patients exposed to corticosteroids as for the entire cohort.

## Discussion

Pediatric ARDS survivors and nonsurvivors have distinct biochemical trajectories over the first 7 days after ARDS onset, with multiple tissue injury biomarkers and DAMPs higher in nonsurvivors. The molecular signature of nonsurvivors overlapped with that of persistent MODS, and less so with persistent ARDS. Important clinical drivers of mortality in pediatric ARDS, such as baseline immune compromise, also had higher levels of tissue injury markers and DAMPs associated with mortality. Overall, we demonstrate that poor outcomes in pediatric ARDS are linked primarily to the hyperinflammatory response, DAMP release, and nonpulmonary organ failure. Collectively, these data suggest that lethality from pediatric ARDS is due to overwhelming systemic inflammation and tissue injury.

There are no successful directed therapies for either pediatric or adult ARDS. In adult ARDS trials, supportive care measures such as lower ventilator pressures and volumes (25), prone positioning (26), and neuromuscular blockade (27) have demonstrated efficacy in randomized trials. Pleiotropic antiinflammatories such as methylprednisolone and dexamethasone may have potential efficacy, as well (28–30). Our results suggest that interventions directed at mitigating progressive organ failures in ARDS is an appropriate target for improving mortality. Notably, endotheliopathy (elevated ANG2) was implicated for both persistent ARDS and for persistent MODS, suggesting this as an attractive targetable pathway for future intervention. We note, however, that dedicated studies testing interventions in pediatric ARDS stratified according to biomarker signature are necessary to fully assess whether a given molecular profile defines an “endotype” or a “treatable trait” (31, 32).

Nonsurvivors consistently demonstrated not just higher overall levels of multiple inflammatory biomarkers, but also trajectories of increasing (primarily) cytokines, proteases, and chemokines. This signal was also seen in sicker patients, including those with baseline immune compromise and those who received corticosteroids. Concurrent development of endothelial damage, as evinced by rising ANG2, was also consistently associated with mortality. Increasing inflammatory and endothelial damage biomarkers have been described in COVID-19 (33, 34), but few studies have investigated longitudinal trajectory in non-COVID ARDS, and none in pediatrics. The significance of these increases is unclear, and while parallel increases in inflammation and endothelial damage are perhaps unsurprisingly associated with worse outcome, the identification that these elevations occur after ARDS onset highlights their potential as therapeutic targets to improve outcomes.

SPD (type II alveolar epithelia) and ANG2 (endothelial cells) increased over the first 7 days in PICU nonsurvivors, whereas sRAGE (scavenger receptor for AGEs expressed highest in type I alveolar epithelia) peaked on day 0 and then decreased in all patients, albeit higher in nonsurvivors at all time points. This suggests that alveolar damage may be a later phenomenon in pediatric ARDS, and that the elevated sRAGE on day 0 may not solely (or primarily) reflect a lung source. Supporting this, SPD was the sole biomarker tested that was higher in direct ARDS; sRAGE was nonsignificantly elevated in indirect ARDS. Despite high sRAGE expression in type I pneumocytes, its exact tissue origin in ARDS is unclear, with some evidence suggesting the endothelium (8, 35, 36) or leukocytes (37) as a significant source. Mendelian randomization has implicated sRAGE as a causal intermediate for ARDS development in septic adults (9), while in adults with hypertension (38) and diabetes (39, 40) sRAGE correlated with endothelial dysfunction and inflammation. We provide additional nuance to existing sRAGE literature by reporting values over the first 7 days of pediatric ARDS, confirming its association with mortality and MODS, and demonstrating temporal kinetics completely distinct from SPD or ANG2.

The later increase in SPD in nonsurvivors may reflect propagation of the immune response in lungs, especially as infectious etiologies (pneumonia and sepsis) were the primary etiologies of ARDS. Adults with ARDS who develop secondary pulmonary bacterial infections have elevated circulating SPD (41), but this was not seen in children (42). Few studies have investigated the longitudinal kinetics of SPD in either adults or pediatrics (33, 43), but increasing levels later after ARDS onset have been reported in adults with COVID-19 (33). Furthermore, SPD has been implicated as a marker of alveolar damage due to ventilator adjustments, such as in patients exposed to higher driving pressure (44), and so later elevations in nonsurvivors could reflect clinician-determined ventilator adjustments. Causality between injury and SPD levels is difficult to extrapolate from our observational cohort, as sicker patients with ongoing alveolar inflammation and injury are plausibly exposed to higher and more damaging ventilator settings. Overall, however, our data support longitudinal SPD measurements in future trials of ventilator settings in pediatric ARDS, with the elevated levels in nonsurvivors suggesting that increases in SPD in response to therapies warrant attention as a possible early surrogate for poor outcomes.

Multiple DAMPs, notably nucleosomes (histone/DNA complexes) and *COX4* (nDNA), were elevated in nonsurvivors. Interestingly, mtDNA was not elevated in nonsurvivors, in contrast with adult data (45–47), demonstrating the necessity of translational studies specifically in children. Levels of mtDNA in our cohort were comparable to adult ARDS cohorts (6). It is possible that mtDNA is not as biologically relevant in this population, and that the degree of organ failure induced, if any, by mtDNA in pediatric ARDS does not impact mortality. As there are multiple mechanisms by which mtDNA is released into circulation (48), including cell death, activated immune cell release, or mitochondrial stress and pore formation, it is possible that children have different etiologies of mtDNA escape relative to adults. Specific studies comparing adult and pediatric modes of mtDNA release and mitochondrial resilience are warranted. Alternatively, it is possible that there are differences in monocyte phenotype between children and adults, specifically TLR9 expression. In critically ill adults, mtDNA was only associated with higher mortality in patients with elevated monocyte TLR9 expression (49).

Notably, not all studies of critically ill adults have confirmed an association between elevated mtDNA and mortality. A recent study of hospitalized adults with COVID-19 found that plasma nDNA, but not mtDNA, predicted mortality (50), consistent with our results. Similarly, in adults with trauma, plasma nDNA was associated with worse outcomes, whereas mtDNA did not predict clinical trajectory (51). Our results extend previous findings in this first report to our knowledge of cell-free DNA (cfDNA) (both nDNA and mtDNA) in pediatric ARDS by confirming the prognostic utility of nDNA.

Our group has previously demonstrated the prognostic utility of nucleosomes on day 0 of pediatric ARDS (52), which we have now confirmed over the first 7 days of ARDS. The strong correlation between nucleosomes and nDNA likely explains the prognostic utility of nDNA in this cohort. Unlike mtDNA, circulating nDNA is generally not considered a DAMP, although one prior study in COVID-19 suggested nDNA could contribute to inflammation via TLR9, similarly to mtDNA (50). Multiple mechanisms have been invoked for release of cfDNA (both nDNA and mtDNA) in critical illness, including apoptosis (53, 54), necrosis (53), necroptosis (55), and NETosis (neutrophil extracellular trap formation) (53, 56). Mechanisms of cell death were not investigated, and the contribution of NETosis, or any other specific form of cell death or cfDNA release, cannot be established. However, plasma nDNA methylomics can be leveraged to identify the cellular origins of cfDNA (57, 58).

Interestingly, the biomarker levels and trajectories associated with nonsurvival were similar when examining the entire cohort and when restricted to those exposed to corticosteroids. It is possible that the doses of corticosteroid used (previously reported in this cohort at median 1 mg/kg methylprednisolone equivalent for a median of 7 days; ref. 24) does not meaningfully affect biomarker levels or trajectories. Alternatively, as corticosteroids were used in patients with worse lung mechanics (Supplemental Table 3), it is possible that the inflammatory and tissue damage signature of severe ARDS overlapped with the signature that was also associated with mortality. However, the nonrandomized nature of this observational study precludes firm conclusions regarding the relationship between ARDS severity, corticosteroid use, biomarker levels, and eventual outcome.

### Limitations.

Our study has limitations. Patients were from a single center, and while clinical characteristics are similar to other cohorts (4, 59), generalizability cannot be assumed. The granular data collected from our center permitted controlling for variables known to affect biomarker levels and outcomes (Supplemental Figure 10), thereby providing a less biased estimate of the association between overall biomarker levels and trajectory over the first 7 days of ARDS with outcomes. We chose PaO_2_/FIO_2_, rather than oxygenation index, as the cohort was selected using Berlin eligibility criteria. The other confounders (age, ARDS etiology, immunocompromised status) were chosen for plausible association with outcome and biomarker levels, and because they represent premorbid confounders. By design, we did not adjust for severity of illness scores or organ failure, as these are quantified after PICU admission and ARDS onset (by definition or by practice), and would potentially be on the causal pathway linking biomarkers with outcome. Thus, as potential mediators, we did not adjust for these.

The sample size was modest, albeit reasonably large for pediatric ARDS, and the power to detect associations between biomarker levels and trajectories identified by regression are partly dependent on the relative rates of the outcomes (e.g., mortality). As further subdivisions risked underpowering our analyses, we did not perform any cross validations. We required an arterial blood gas for enrollment and may have missed patients with ARDS lacking a diagnostic PaO_2_. A study applying pediatric-specific definitions (60) using oxygenation index and less restrictive radiographic criteria would possibly have different conclusions. However, we chose to use the 2012 Berlin definition of ARDS (2), rather than the 2015 Pediatric Acute Lung Injury Consensus Conference (PALICC) definition (60), because the requirement for bilateral opacities in Berlin represented an established and more specific definition of ARDS. Accordingly, all but one patient in our cohort met PALICC criteria for pediatric ARDS.

Additionally, the use of peripheral blood, rather than the alveolar compartment, may have enriched for biomarker signatures of tissue injury and inflammation instead of lung injury. Using previously published methods (47), we report on 2 amplicons for mtDNA and a single amplicon for nDNA, yielding measurements of mtDNA that correlate with one another and measurements of nDNA that correlate with nucleosome levels, suggesting internal consistency and confidence in these results. Future studies using broader coverage of mtDNA and nDNA with additional amplicons or orthogonal methods may prove informative.

Finally, fewer patients were available on days 3 and 7 for biomarker analysis, leading to bias from informative dropout. The directionality of this potential bias is unpredictable, as early nonsurvivors and rapid improvers may have affected the association either upward or downward between a biomarker and outcome had there been available plasma on day 7. However, we are reassured that results did not change when considering only complete cases. Future studies are warranted to extend these findings in larger multicenter cohorts, with particular focus on changes in nDNA levels and tissue origins of circulating DAMPs over the time course of pediatric ARDS.

### Conclusions.

In a longitudinal comprehensive biomarker profiling study, pediatric ARDS survivors and nonsurvivors demonstrated distinct biomarker trajectories, with nonsurvivors showing elevations in inflammatory cytokines, tissue injury markers, and DAMPs. There was strong overlap between nonsurvivors and persistent MODS. Collectively, these findings suggest that DAMP signaling and ongoing endothelial and tissue damage appear to be the dominant pathology contributing to mortality and organ failure in pediatric ARDS. Consequently, exploring global endothelial dysregulation and DAMP release in ARDS may illuminate novel mechanisms and identify targetable pathways for this devastating syndrome.

## Methods

### Sex as a biological variable.

Male and female patients were included in this study, and 44% of the cohort is female.

### Study design and patient selection.

This was a prospective cohort study of children with Berlin-defined (2) ARDS enrolled at the Children’s Hospital of Philadelphia (CHOP) between July 2015 and December 2019. The overall aim of this cohort study was to associate select biomarkers with clinical outcomes (Supplemental Table 1), with a pilot phase of sample collection only on day 0 (≤24 hours of ARDS onset) (61, 62) and a subsequent longitudinal phase with sample collection on days 0, 3, and 7. Portions of this cohort using day 0 samples have been previously described (52, 63, 64); however, the longitudinal cohort has not been previously reported.

PICU patients were screened daily. Inclusion criteria were (a) acute respiratory failure requiring invasive ventilation; (b) arterial access; (c) age greater than 1 month and less than 18 years; (d) 2 consecutive PaO_2_/FIO_2_ of 300 or less, 1 or more hours apart, on positive end-expiratory pressure (PEEP) of 5 cmH_2_O or greater; and (e) bilateral infiltrates separately adjudicated by a radiologist and intensivist. Exclusion criteria were (a) respiratory failure primarily from cardiac failure, (b) chronic respiratory disease, (c) ventilator dependence, (d) cyanotic heart disease, (e) ventilation for more than 7 days before PaO_2_/FIO_2_ of 300 or less, and (f) ARDS established outside of the CHOP PICU.

### Definitions.

Biomarkers were the primary exposure. PICU mortality was the primary outcome. We also assessed the outcomes of persistent ARDS (PaO_2_/FIO_2_ ≤ 200 on day 7) and persistent MODS (at least 2 nonpulmonary organ failures on day 7).

ARDS was characterized as direct (primarily pulmonary) and indirect (primarily nonpulmonary). Infectious pneumonia, aspiration, drowning, pulmonary contusion, and smoke inhalation were considered direct ARDS; nonpulmonary sepsis, nonthoracic trauma, noncardiogenic shock, transfusion-related acute lung injury, and pancreatitis were indirect. Etiology was determined primarily by chart abstraction by trained study personnel in discussion with the attending physician on the likely etiology. Uncertain cases were adjudicated by a 3-person team of PICU physicians, with discussion until unanimous consensus. Assignment of patients to hypo- and hyperinflammatory ARDS subphenotypes was performed using parsimonious algorithms described for this cohort (65).

Metrics of oxygenation utilized were PaO_2_/FIO_2_ and oxygenation index, calculated as (mean airway pressure × FIO_2_ × 100)/PaO_2_. Shock severity was quantified with the vasopressor score (66, 67). Nonpulmonary organ failures (neurologic, cardiovascular, hematologic, renal, hepatic) at ARDS onset were identified using pediatric sepsis definitions (68). Severity of illness was quantified using the 12-hour PRISM III score (69). The designation “immunocompromised” required presence of an immunocompromising diagnosis (oncologic, immunologic, rheumatologic, transplant) and active immunosuppressive therapy, or presence of a congenital immunodeficiency (70, 71). Prospective data collection included ventilator settings and gas exchange at ARDS onset, ancillary ARDS therapies used in the first 3 days, and any escalation to extracorporeal membrane oxygenation (ECMO).

### Plasma collection and protein biomarker measurements.

Blood was collected in citrated tubes at 3 time points: within 24 hours of ARDS onset (time of meeting all Berlin criteria: day 0), on day 3, and on day 7. Samples were centrifuged (2000*g*, 20 minutes, 20°C) within 30 minutes of sample collection, aliquoted to prevent freeze/thaw cycles, and stored at –80°C until analysis.

Biomarkers were measured using a combination of single- and multiplex enzyme-linked immunosorbent assays (ELISAs) at CHOP unless otherwise specified. Granzyme B, HSP70, IL-1α, IL-8, CCL3/MIP-1α, MIP-1β, and MMP8 were measured on a Luminex platform (63) at Cincinnati Children’s Hospital Medical Center. CCL7, CCL22, IL-6, sTNFR1, and TNF-α were measured using a custom Ella (Biotechne) multiplex at Penn State Hershey. Nucleosomes were measured using a singleplex ELISA (Sigma-Aldrich). P3NP (Abbexa), ANG2, sRAGE, and SPD were measured using singleplex ELISAs (others all R&D Systems). Overall variability was minimal between plates (standard deviation [SD]/mean < 15%), with samples above and below lower limits of detection set to equal the highest or lowest value for that plate. All analytes were measured in duplicate irrespective of platform, with minimal variability (SD/mean < 10%).

### cfDNA measurements.

Circulating cfDNA was extracted from plasma using the DNeasy Blood and Tissue kit (Qiagen). DNA levels for each sample were quantified in triplicate using LightCycler Fast Start DNA Master SYBR green I (Roche) and QuantStudio 7 (Applied Biosystems). The following primers were used: *ND1*: 5′-ATACCCATGGCCAACCTCCT-3′ and 5′-GGGCCTTTGCGTAGTTGTAT-3; *COX1*: 5′-TGATCTGCTGCAGTGCTCTGA-3′ and 5′-TCAGGCCACCTACGGTGAA-3′; *COX4*: 5′-GAAAGTGTTGTGAAGAGCGAAGAC-3′ and 5′-GTGGTCACGCCGATCCAT-3′.

The PCR standard for *COX1* was a gift from Neal Sondheimer (University of Toronto). PCR standards for *ND1* and *COX4* were amplified from DNA extracted from endothelial cell lysates and gel purified using a QiaEX II Gel Extraction Kit (Qiagen). Copy number per microliter of samples was calculated using an online copy number calculator at https://scienceprimer.com/copy-number-calculator-for-realtime-pcr

### Statistics.

Analyses were performed with Stata/MP 18 (https://www.stata.com/statamp/). Clinical data are reported as median and IQR, and differences between groups compared using nonparametric statistics. All biomarkers were log transformed for downstream analyses.

To assess relationships between biomarkers, we computed Pearson’s correlation coefficients separately for biomarkers on days 0, 3, and 7. Additionally, we performed hierarchical clustering (Euclidean distance, complete linkage, for both biomarkers and patients) to assess whether patterns of correlated biomarkers clustered with ARDS subphenotypes and with mortality, repeating the analyses separately for days 0, 3, and 7. We then assessed the relationship between log-transformed biomarkers and PICU mortality on days 0, 3, and 7 adjusted for confounders chosen a priori: age, ARDS etiology, immunocompromised status, and PaO_2_/FIO_2_ at ARDS onset. We intentionally did not include metrics of severity of illness (PRISM III, vasopressor score, organ failures), as they were potential mediators of the association between biomarkers and mortality (Supplemental Figure 10). These analyses were adjusted for multiple corrections using Bonferroni’s test (unadjusted *P* < 0.0025; corrected *P* < 0.05).

Finally, we assessed differences in biomarker level and trajectory over the first 7 days between PICU survivors and nonsurvivors using mixed effects regression, adjusting for the same confounders as above. To facilitate comparisons between biomarkers of different scale, log-transformed biomarker values were also standardized (mean = 0, SD = 1). Similar analyses were conducted to assess differences between patients with and without persistent ARDS by day 7, with and without persistent MODS by day 7, between patients classified as direct or indirect ARDS, between patients classified as immunocompetent or immunocompromised, and between patients exposed or unexposed to systemic corticosteroids. As immune status and corticosteroid use can impact biomarker trajectories, we also assessed the association between biomarker levels and trajectory over the first 7 days of ARDS between PICU survivors and nonsurvivors stratified according to immune status and corticosteroid use. In these analyses, we report the difference in the overall biomarker level (over all time points) between groups and differences in biomarker trajectory between groups. We chose to report the overall biomarker level specifically to assess associations with outcomes throughout the first week of ARDS, postulating that this is potentially more informative than examining single time points in isolation. We adjusted for confounders, as others have done in similar analyses (34), in order to present an estimate of the association between a biomarker and its trajectory, with outcomes unconfounded by ARDS severity or etiology.

### Study approval.

The study was approved by the CHOP Institutional Review Board (IRB 13-010578), and informed consent was obtained from caregivers prior to any study procedures.

### Data availability.

Primer sequences used for cfDNA measurements and commercial ELISAs are detailed in Methods. Deidentified patient data are available as part of this manuscript submission (Supporting Data Values file), and can also be obtained upon request from the corresponding author (NY; yehyan@chop.edu), pursuant to regional legal and regulatory constraints. This is most commonly done with the execution of a data use agreement (DUA) between CHOP and the requesting institution for deidentified (no identifiable health information) data. Statistical code is available as Supplemental Data Set 1.

## Author contributions

NY, JDC, and NSM conceived of and designed the study. JMT, DJK, ESH, PL, and BMV were responsible for protein biomarker measurements and analyses. LKML and NSM were responsible for cfDNA measurements and analysis. JET, WZ, and ELC assisted with cfDNA analyses. TJB, GDA, MVM, and GK were responsible for clinical data collection and analyses. NY oversaw all analyses, and serves as guarantor for this manuscript.

## Supplementary Material

Supplemental data

ICMJE disclosure forms

Supplemental data set 1

Supporting data values

## Figures and Tables

**Table 1:**
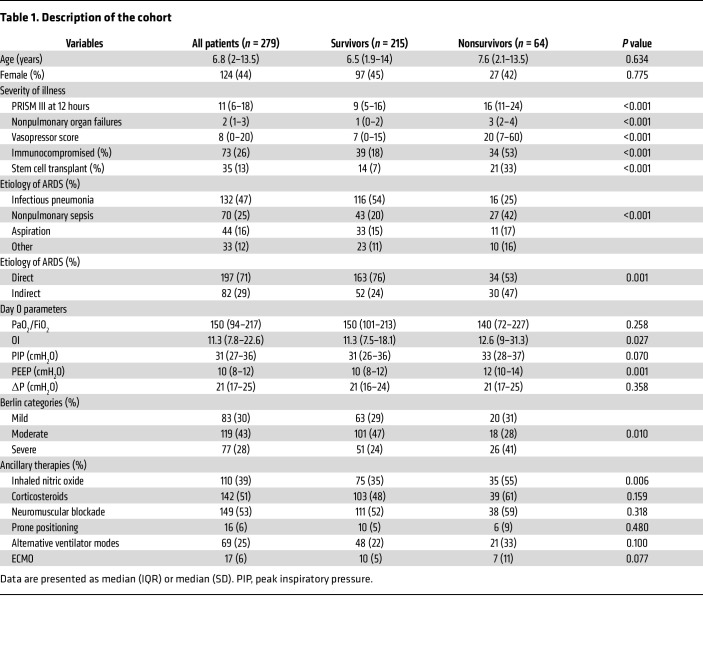
Description of the cohort

## Version 1 04/04/2024

In-Press Preview

## Version 2 05/15/2024

Electronic publication
