# Supplementary material for: Inflammatory and tissue injury marker dynamics in pediatric acute respiratory distress syndrome
Source: J Clin Invest. 2024 Apr 4;134(10):e177896. doi: 10.1172/JCI177896 (PMC11093602; doi:10.1172/JCI177896)

Inflammatory and tissue injury marker dynamics in pediatric acute respiratory distress syndrome

DATA SUPPLEMENT

SECTION	PAGE
**SUPPLEMENTARY TABLES**	
**1:** Biomarker list	2
**2:** Cohort stratified by immunocompromised status	3
**3:** Cohort stratified by corticosteroid use	4
**SUPPLEMENTARY FIGURES**	
**1:** Clinical trajectories of specific organ failures	5
**2:** Hierarchical clustering	7
**3:** Biomarker levels and trajectories associated with mortality (complete cases)	8
**4:** Select biomarker levels	9
**5:** Biomarker levels and trajectories associated with direct/indirect ARDS	10
**6:** Biomarker levels and trajectories associated with immunocompromised status	11
**7:** Biomarker levels and trajectories associated with mortality (by immune status)	12
**8:** Biomarker levels and trajectories associated with corticosteroid use	13
**9:** Biomarker levels and trajectories associated with mortality (by corticosteroid use)	14
**10:** Directed acyclic graph	15

Supplementary Table 1: Biomarkers

Marker	Assay	Comments
**Inflammatory cytokines**		
IL-1α	Luminex	• Non-specific pro-inflammatory cytokine
IL-6, sTNFR1	ELLA	• Non-specific pro-inflammatory cytokines<br>• Discriminates ARDS subphenotypes
**Proteases**		
MMP8	Luminex	• Neutrophil collagenase<br>• Also released by some tissues
Granzyme B	Luminex	• Pro-apoptotic serine protease released by NK cells and cytotoxic T lymphocytes<br>• Also released by some tissues, including type-II alveolar epithelial cells
**Chemokines**		
IL-8	Luminex	• Neutrophil attractant
CCL7	ELLA	• Chemoattractant for multiple leukocytes, including monocytes, NK cells, and activated T lymphocytes
CCL22	ELLA	• Chemoattractant for multiple leukocytes, including monocytes, NK cells, and Th2 lymphocytes<br>• Macrophage-derived
MIP-1α, MIP-1β	Luminex	• Chemoattractant for multiple leukocytes, including monocytes, NK cells, and Th1 lymphocytes
**Tissue injury markers**		
Angiopoietin-2	ELISA	• Endothelial injury marker
P3NP	ELISA	• Marker of collagen turnover and fibrosis
Soluble RAGE	ELISA	• Pro-inflammatory scavenger receptor for AGEs<br>• Expressed in high levels in type-I alveolar epithelia<br>• Pleiotropic expression in endothelium and leukocytes
Surfactant protein D	ELISA	• Type-II alveolar epithelial cell marker
**DAMPs**		
Heat shock protein 70	Luminex	• Released during cell death and can propagate inflammation via TLR2 and TLR4
Nucleosomes	ELISA	• DNA/histone complexes released during cell death with histones acting as DAMPs
COX4 (nuclear DNA)	RT-PCR	• Unclear if directly a DAMP or whether a bystander released alongside histones and nucleosomes
COX1, ND1 (mtDNA)	RT-PCR	• Released during cell death and can propagate inflammation via TLR9

Supplementary Table 2: Cohort stratified by immunocompromised status.

Variables	All Patients (n = 279)	Immuno-competent (n = 206)	Immuno-compromised (n = 73)	P value
Age (years)	6.8 [2, 13.5]	6.3 [1.6, 13.1]	7.1 [3.3, 14.4]	0.078
Female (%)	124 (44)	93 (45)	31 (42)	0.784
Severity of illness				
PRISM III at 12h	11 [6, 18]	10 [5, 17]	14 [8, 19]	0.072
Non-pulmonary organ failures	2 [1, 3]	1 [1, 2]	2 [2, 3]	< 0.001
Vasopressor score	8 [0, 20]	8 [0, 17]	10 [0, 40]	0.127
Stem cell transplant (%)	35 (13)	-	35 (48)	-
Etiology of ARDS (%)				
Infectious pneumonia	132 (47)	104 (50)	28 (38)	
Non-pulmonary sepsis	70 (25)	40 (19)	30 (41)	0.002
Aspiration	44 (16)	38 (18)	6 (8)	
Other	33 (12)	24 (12)	9 (12)	
Day 0 parameters				
PaO_2/FiO_2	150 [94, 217]	146 [88, 213]	156 [118, 220]	0.247
OI	11.3 [7.8, 22.6]	12.1 [7.6, 23.9]	10.1 [8.2, 15.9]	0.372
PIP (cmH_2O)	31 [27, 36]	31 [26, 36]	32 [27, 37]	0.624
PEEP (cmH_2O)	10 [8, 12]	10 [8, 12]	10 [8, 12]	0.927
ΔP (cmH_2O)	21 [17, 25]	21 [16, 25]	21 [17, 24]	0.578
Ancillary therapies (%)				
Inhaled nitric oxide	110 (39)	79 (38)	31 (42)	0.578
Corticosteroids	142 (51)	92 (45)	50 (68)	0.001
Neuromuscular blockade	149 (53)	108 (52)	41 (56)	0.682
Prone positioning	16 (6)	11 (5)	5 (7)	0.573
Alternative ventilator modes	69 (25)	53 (26)	16 (22)	0.636
ECMO	17 (6)	9 (4)	8 (11)	0.082
PICU mortality (%)	64 (23)	30 (15)	34 (47)	< 0.001

Supplementary Table 3: Cohort stratified by corticosteroid use.

Variables	All Patients (n = 279)	No corticosteroids (n = 137)	Corticosteroids (n = 142)	P value
Age (years)	6.8 [2, 13.5]	6.9 [2, 13.1]	6.3 [2, 14]	0.986
Female (%)	124 (44)	53 (39)	71 (50)	0.071
Severity of illness				
PRISM III at 12h	11 [6, 18]	11 [6, 18]	12 [6, 18]	0.597
Non-pulmonary organ failures	2 [1, 3]	2 [1, 2]	2 [1, 3]	0.854
Vasopressor score	8 [0, 20]	8 [0, 15]	10 [0, 25]	0.229
Immunocompromised (%)	73 (26)	23 (17)	50 (35)	0.001
Stem cell transplant (%)	35 (13)	11 (8)	24 (17)	0.030
Etiology of ARDS (%)				
Infectious pneumonia	132 (47)	55 (40)	77 (54)	
Non-pulmonary sepsis	70 (25)	33 (24)	37 (26)	0.015
Aspiration	44 (16)	26 (19)	18 (13)	
Other	33 (12)	23 (17)	10 (7)	
Day 0 parameters				
PaO_2/FiO_2	150 [94, 217]	155 [101, 230]	146 [83, 197]	0.067
OI	11.3 [7.8, 22.6]	10.1 [6.8, 17.6]	12.9 [8.7, 25.6]	0.002
PIP (cmH_2O)	31 [27, 36]	29 [26, 34]	34 [30, 38]	< 0.001
PEEP (cmH_2O)	10 [8, 12]	10 [8, 12]	10 [10, 12]	0.029
ΔP (cmH_2O)	21 [17, 25]	19 [16, 22]	22 [18, 26]	< 0.001
Ancillary therapies (%)				
Inhaled nitric oxide	110 (39)	40 (29)	70 (49)	0.001
Neuromuscular blockade	149 (53)	60 (44)	89 (63)	0.002
Prone positioning	16 (6)	5 (4)	11 (8)	0.198
Alternative ventilator modes	69 (25)	30 (22)	39 (27)	0.332
ECMO	17 (6)	5 (4)	12 (8)	0.273
PICU mortality (%)	64 (23)	25 (18)	39 (27)	0.087

Supplementary Figure 1: Specific organ failure trajectories (Goldstein definitions). Organ failures status from day 0 to day 3 are stratified according to whether the organ was failing (aqua) or not (olive). Similarly, trajectories between days 3 and 7 are stratified according to whether organs were failing on day 3 (red) or not (blue). By day 7, 45 subjects had been discharged alive from the PICU, and 27 had died. Note that these 27 non-survivors within 7 days of ARDS onset represent a subset of the total (n = 64) that died in the PICU.

Cardiovascular

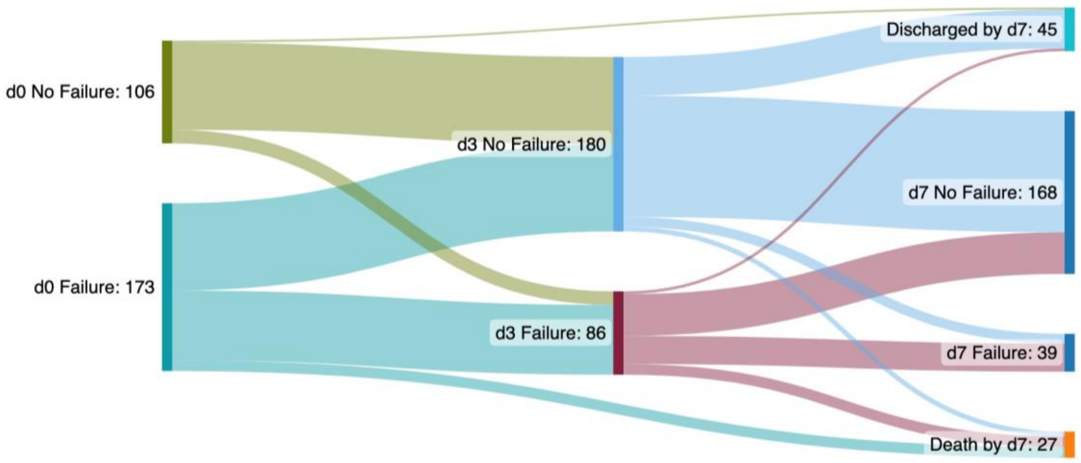

Renal

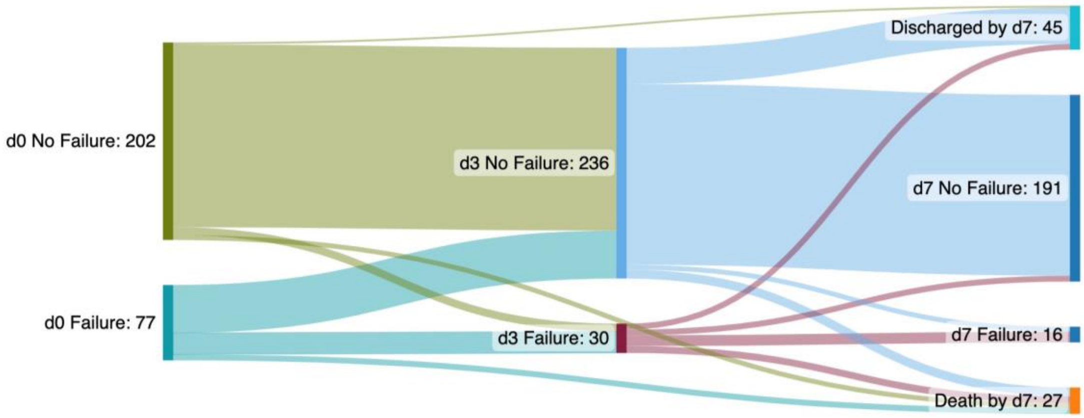

Liver

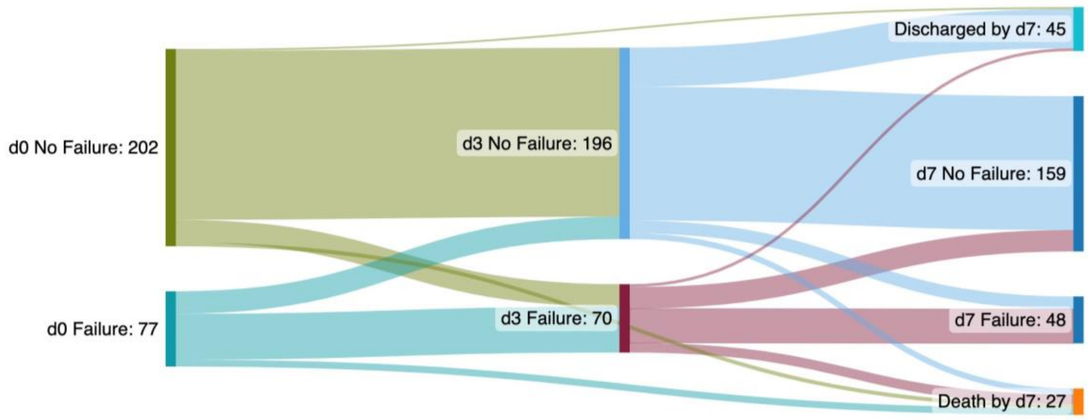

Hematologic

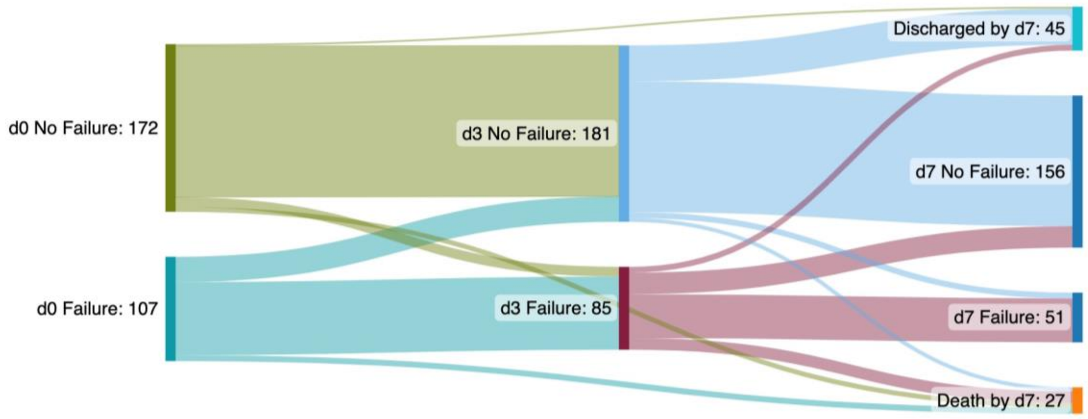

Neurologic

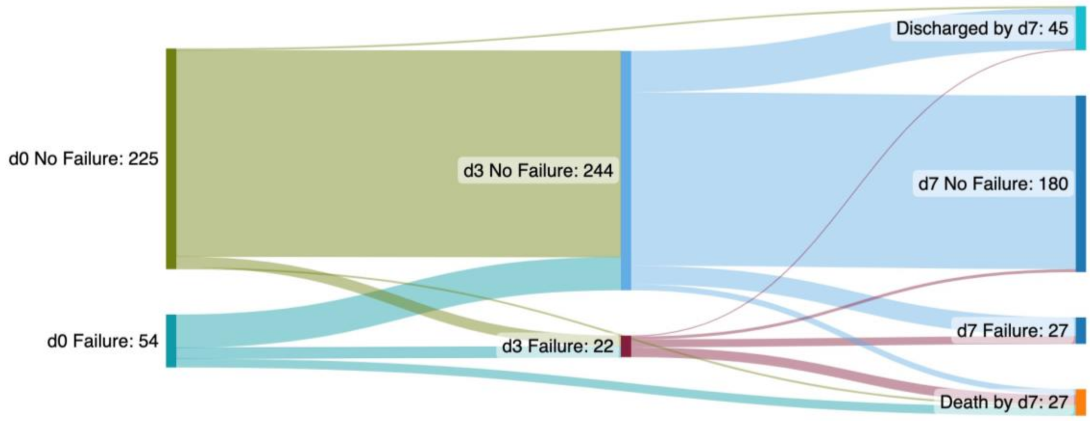

Supplementary Figure 2: Hierarchical clustering of biomarkers and subjects on days 0, 3, and 7 after ARDS onset. DAMPs, tissue injury markers, and cytokines cluster together on days 0 and 3. Hyperinflammatory ARDS and non-survivors are enriched within these signatures.

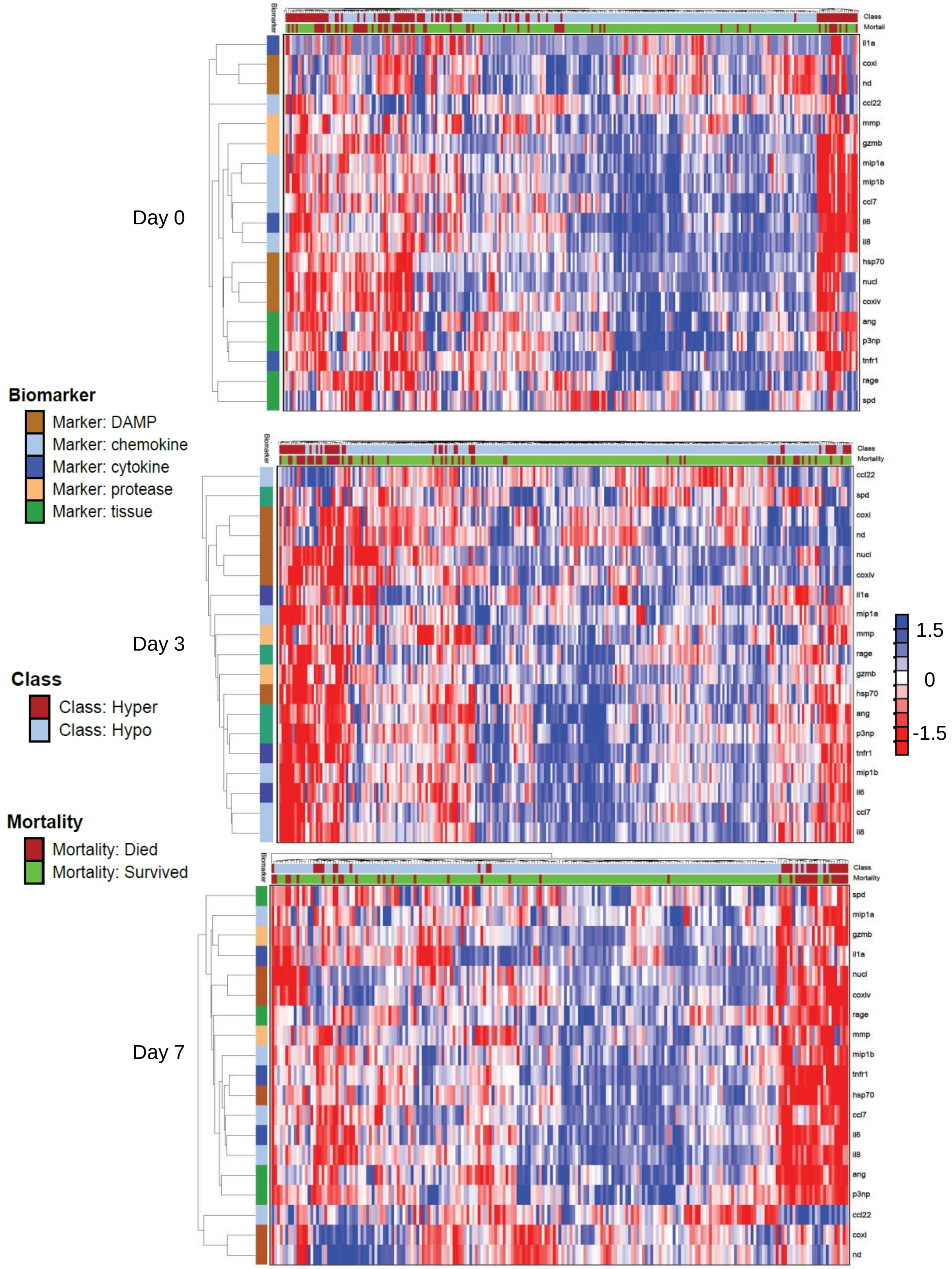

Supplementary Figure 3: Association between biomarker levels and trajectory over the first 7 days of ARDS with PICU mortality restricted to subjects alive and in the PICU for all three timepoints (n = 207). Beta coefficients (and 95% confidence intervals) are plotted for the association between the overall biomarker level in the first 7 days of ARDS and the trajectory with PICU mortality. Biomarker levels are log-transformed and standardized (set to mean = 0, SD = 1), and then adjusted for age, ARDS etiology, immunocompromised status, and initial PaO2/FIO2 in a multivariable mixed effects model. Red dots represent biomarkers with adjusted p < 0.05 with higher levels in non-survivors, blue dots represent biomarkers with adjusted p < 0.05 with lower levels in non-survivors, and black dots represent those with p > 0.05.

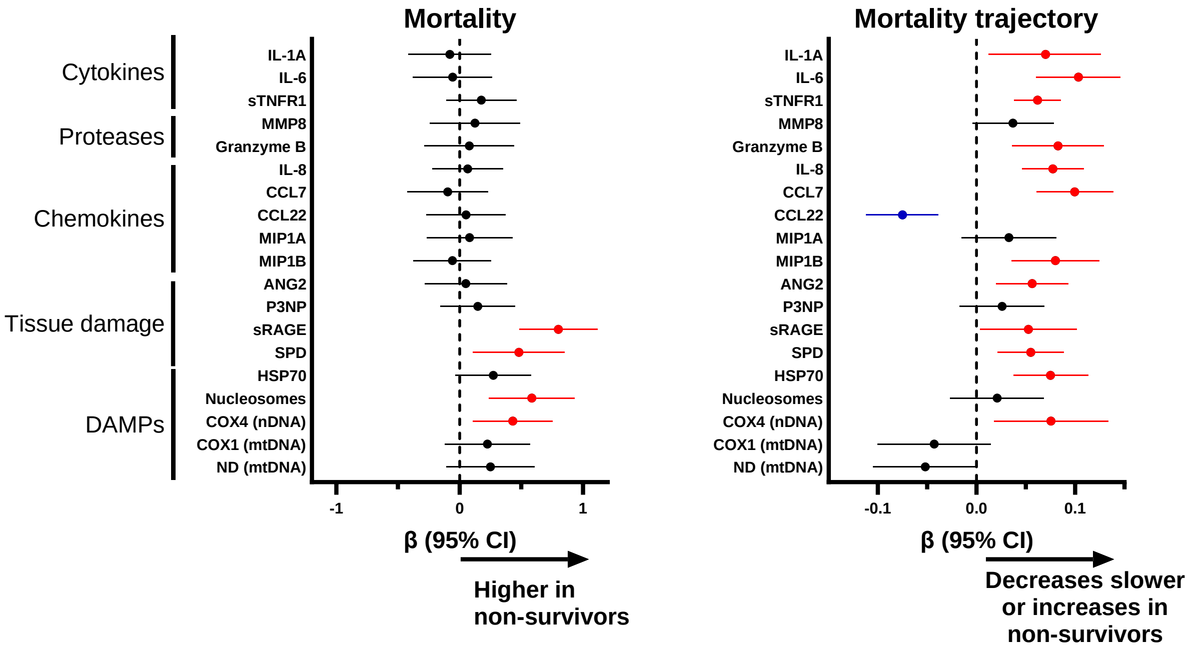

Supplementary Figure 4: Unadjusted plasma biomarker levels between survivors (blue) and non-survivors (red) on days 0, 3, and 7 of ARDS. Black bars are median values. Unadjusted Wilcoxon rank sum tests compare survivors and non-survivors on days 0, 3, and 7 (*: p < 0.05; **: p < 0.01; ***: p < 0.001). Select biomarkers are shown, with the remainder in main Figure 6.

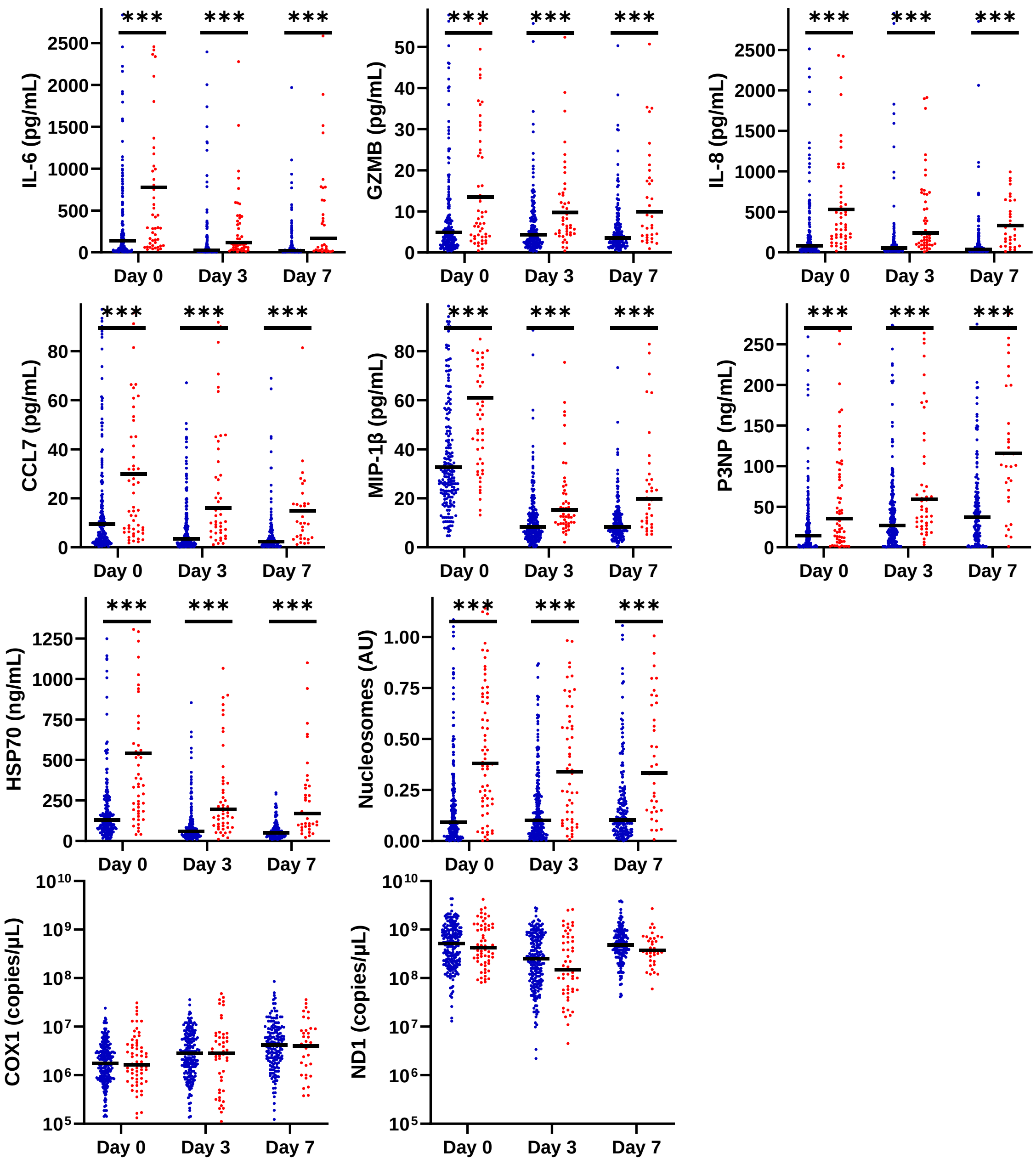

Supplementary Figure 5: Association between biomarker levels and trajectory over the first 7 days of ARDS with direct versus indirect ARDS. Beta coefficients (and 95% confidence intervals) are plotted for the association between the overall biomarker level in the first 7 days of ARDS and the trajectory with indirect ARDS (baseline = direct ARDS). Biomarker levels are log-transformed and standardized (set to mean = 0, SD = 1), and then adjusted for age, immunocompromised status, and initial PaO2/FIO2 in a multivariable mixed effects model. Red dots represent biomarkers with adjusted p < 0.05 with higher levels in indirect ARDS, blue dots represent biomarkers with adjusted p < 0.05 with lower levels in indirect ARDS, and black dots represent those with p > 0.05.

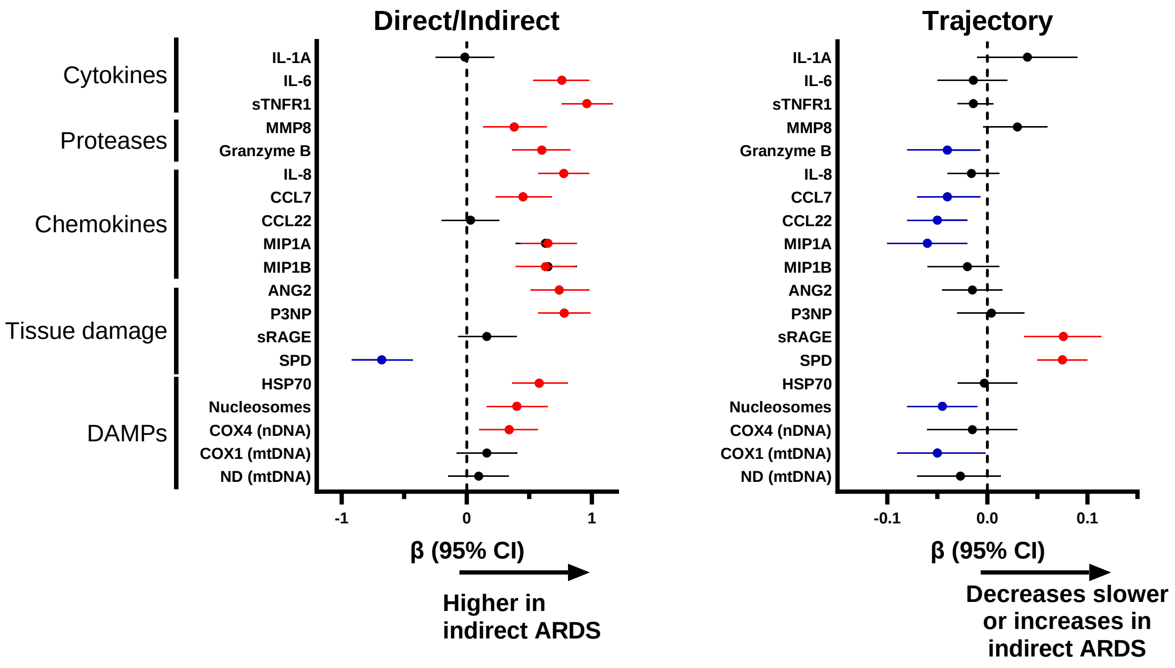

Supplementary Figure 6: Association between biomarker levels and trajectory over the first 7 days of ARDS with immunocompromised status. Beta coefficients (and 95% confidence intervals) are plotted for the association between the overall biomarker level in the first 7 days of ARDS and the trajectory with immunocompromised status (baseline = immunocompetent). Biomarker levels are log-transformed and standardized (set to mean = 0, SD = 1), and then adjusted for age, ARDS etiology, and initial PaO2/FIO2 in a multivariable mixed effects model. Red dots represent biomarkers with adjusted $p < 0.05$ with higher levels in immunocompromised subjects, blue dots represent biomarkers with adjusted $p < 0.05$ with lower levels in immunocompromised subjects, and black dots represent those with $p > 0.05$.

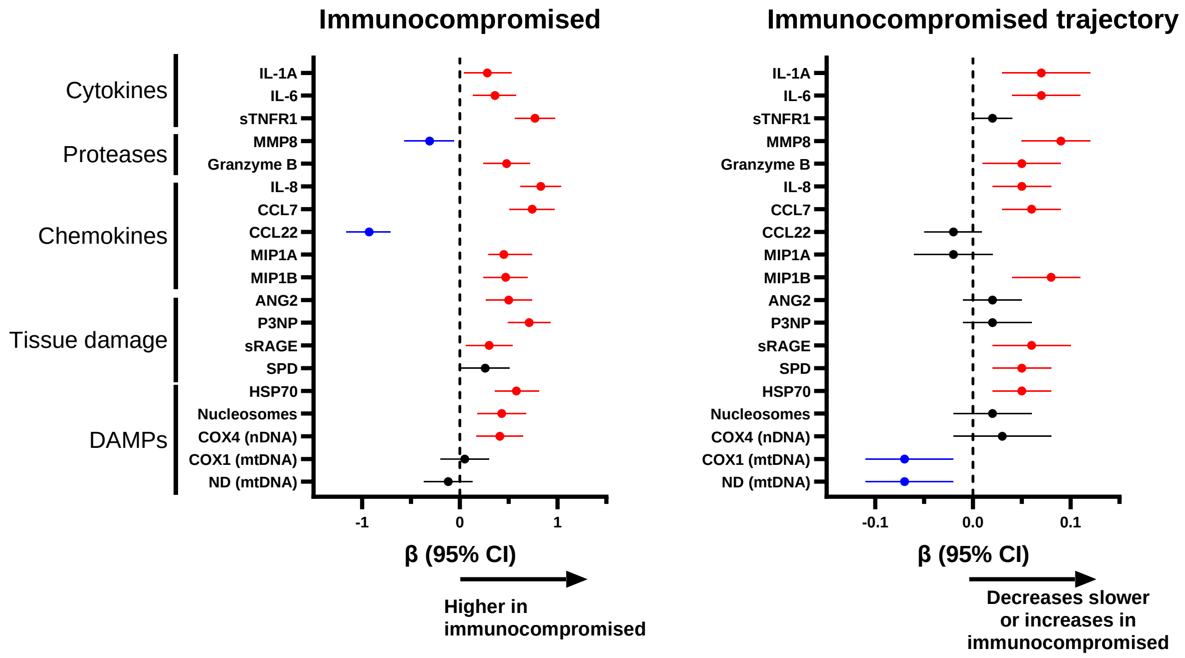

Supplementary Figure 7: Association between biomarker levels and trajectory over the first 7 days of ARDS with PICU mortality, stratified by baseline immune status (immunocompetent or immunocompromised). Beta coefficients (and 95% confidence intervals) are plotted for the association between the overall biomarker level in the first 7 days of ARDS with PICU mortality. Biomarker levels are log-transformed and standardized (set to mean = 0, SD = 1), and then adjusted for age, ARDS etiology, immunocompromised status, and initial PaO2/FIO2 in a multivariable mixed effects model. Red dots represent biomarkers with adjusted $p < 0.05$ with higher levels in non-survivors, blue dots represent biomarkers with adjusted $p < 0.05$ with lower levels in non-survivors, and black dots represent those with $p > 0.05$.

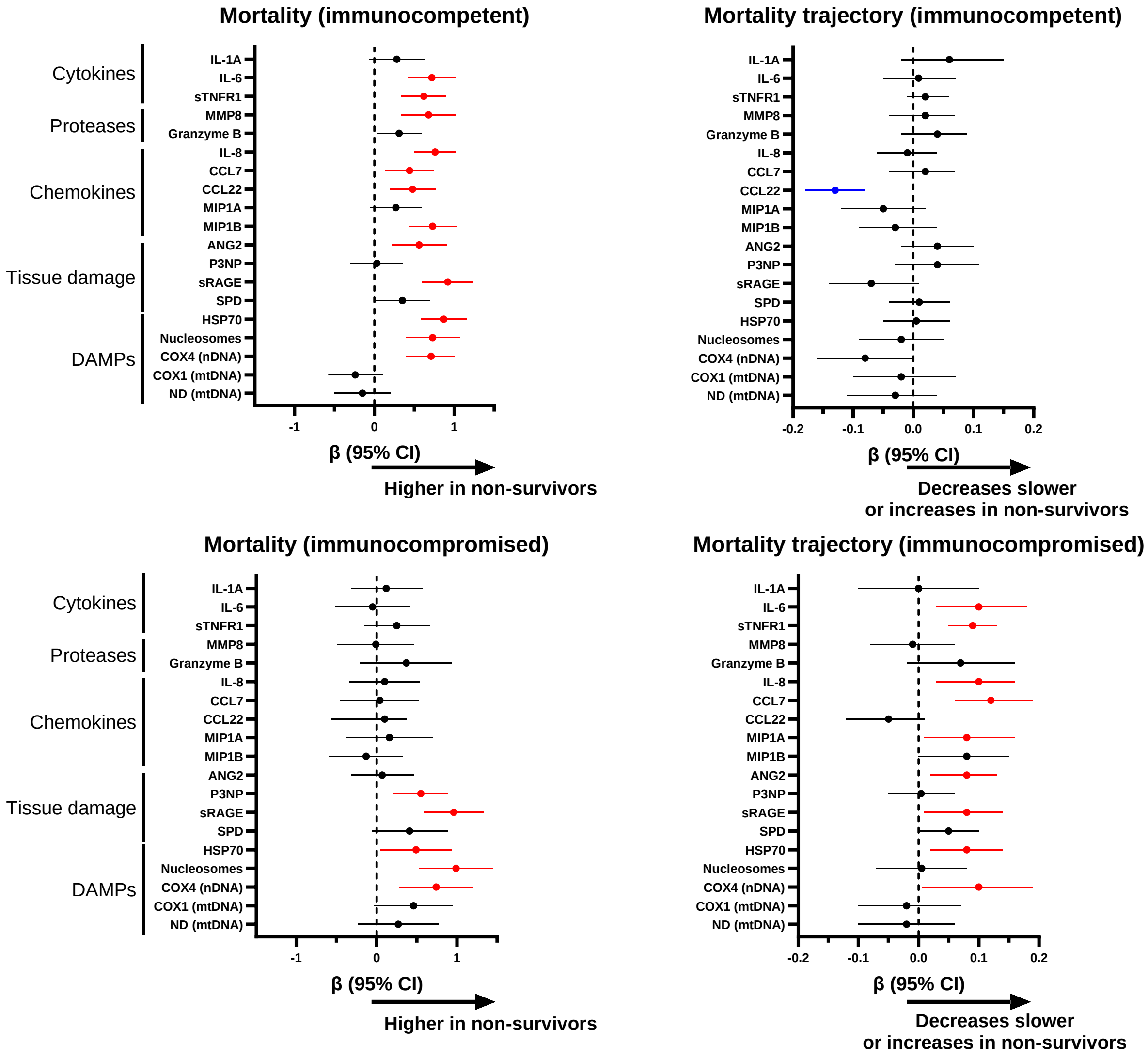

Supplementary Figure 8: Association between biomarker levels and trajectory over the first 7 days of ARDS with exposure to corticosteroids within the first 3 days of ARDS. Beta coefficients (and 95% confidence intervals) are plotted for the association between the overall biomarker level in the first 7 days of ARDS and the trajectory with corticosteroid use (baseline = no corticosteroid use). Biomarker levels are log-transformed and standardized (set to mean = 0, SD = 1), and then adjusted for age, ARDS etiology, immunocompromised status, and initial PaO2/FIO2 in a multivariable mixed effects model. Red dots represent biomarkers with adjusted $p < 0.05$ with higher levels in the corticosteroid group, blue dots represent biomarkers with adjusted $p < 0.05$ with lower levels in the corticosteroid group, and black dots represent those with $p > 0.05$.

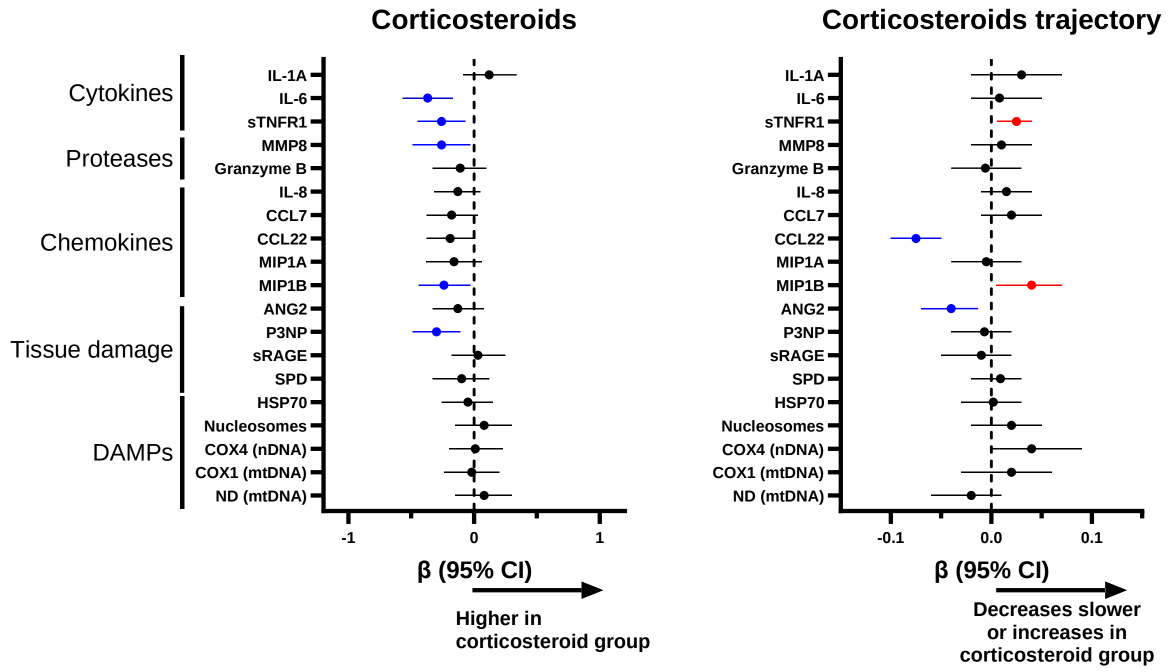

Supplementary Figure 9: Association between biomarker levels and trajectory over the first 7 days of ARDS with PICU mortality, stratified by corticosteroid use within the first 3 days of ARDS. Beta coefficients (and 95% confidence intervals) are plotted for the association between the overall biomarker level in the first 7 days of ARDS with PICU mortality. Biomarker levels are log-transformed and standardized (set to mean = 0, SD = 1), and then adjusted for age, ARDS etiology, immunocompromised status, and initial PaO2/FIO2 in a multivariable mixed effects model. Red dots represent biomarkers with adjusted $p < 0.05$ with higher levels in non-survivors, blue dots represent biomarkers with adjusted $p < 0.05$ with lower levels in non-survivors, and black dots represent those with $p > 0.05$.

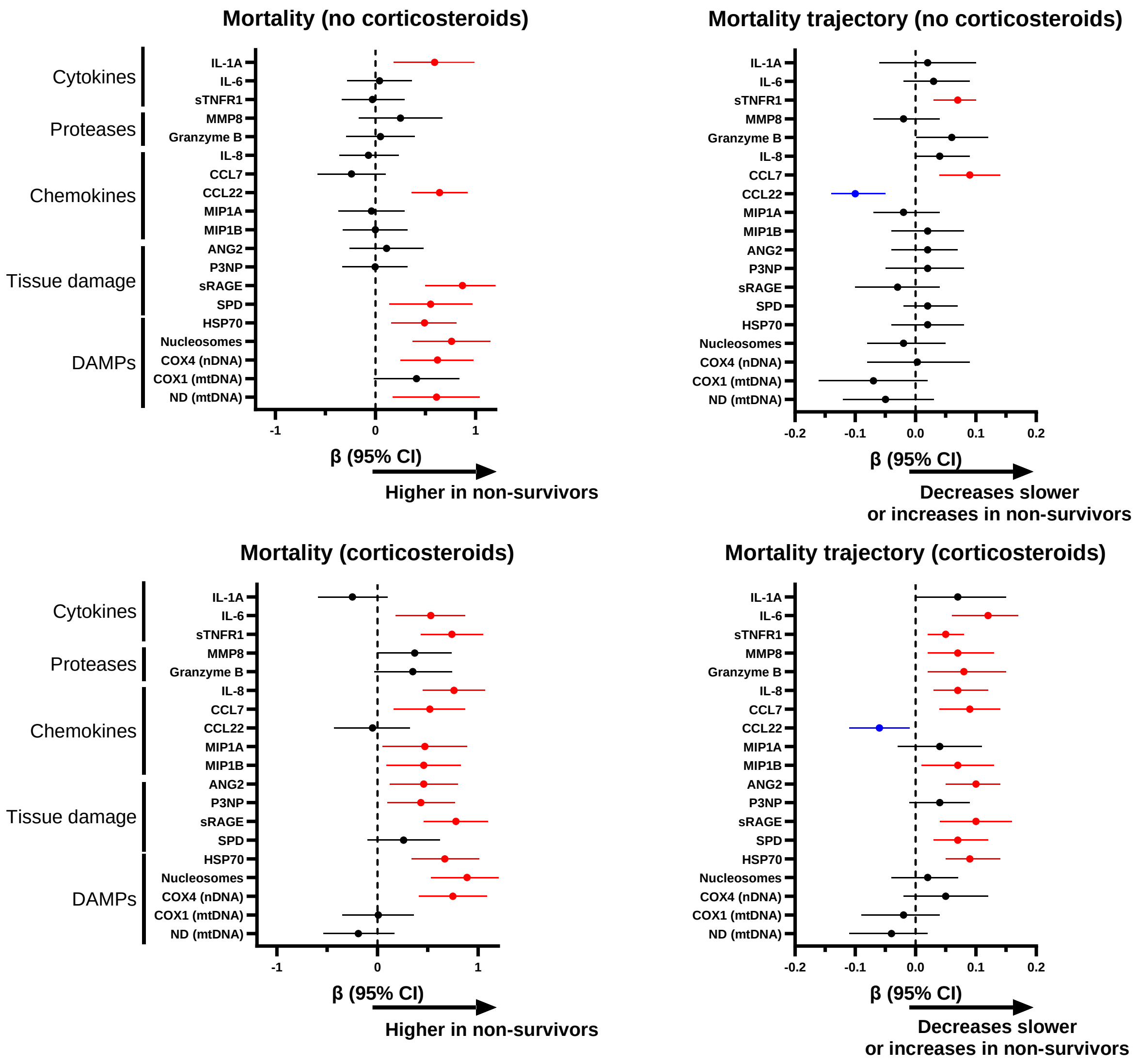

Supplementary Figure 10: Directed acyclic graph (DAG) depicting the relationship between the exposures (biomarkers) and the outcome (PICU mortality, persistent ARDS, or persistent MODS). Note that the confounders (red) that were adjusted for in regression models were chosen for plausible upstream associations with both the exposure (biomarker) and the eventual outcomes (e.g., mortality). Mediators (blue) were not adjusted for as they were thought to mediate the association between the biomarker and outcomes along a causal pathway.

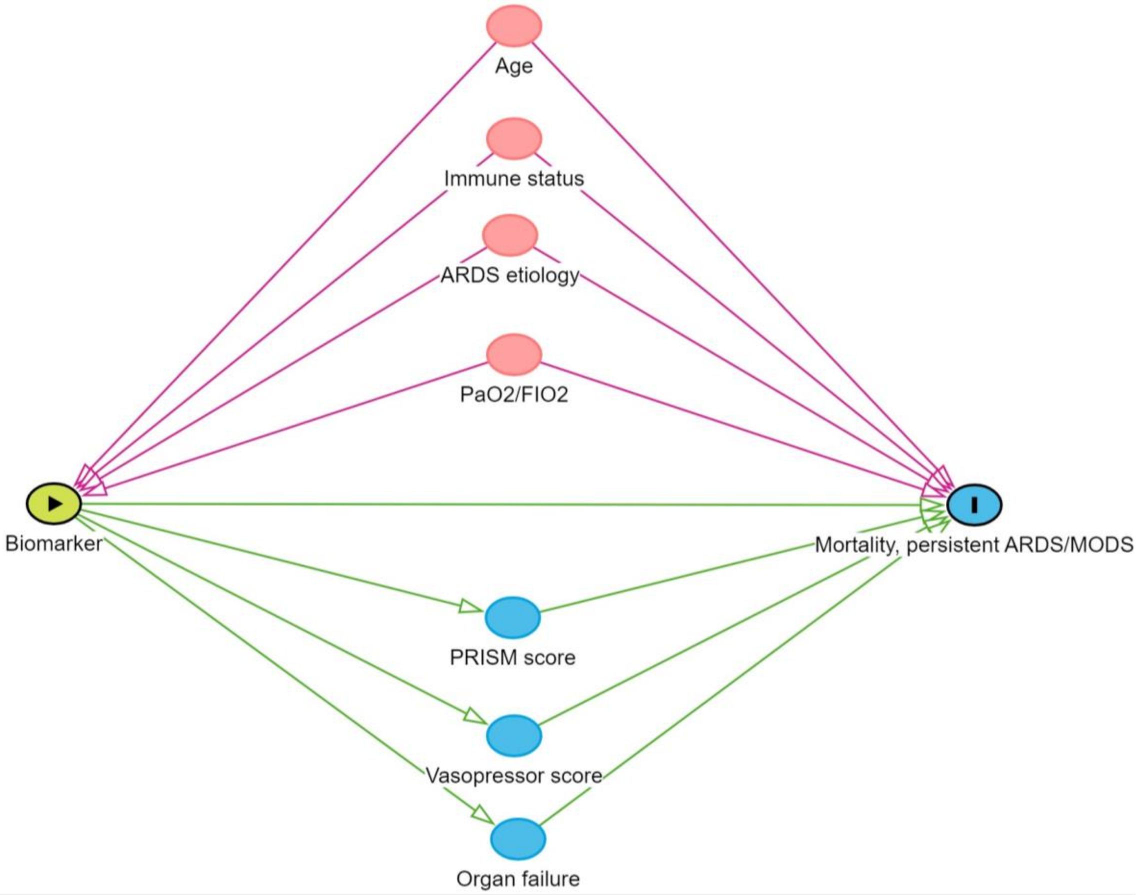

Supplement: Supplemental data [file jci-134-177896-s009.pdf]
