# Supplementary material for: Inflammatory and tissue injury marker dynamics in pediatric acute respiratory distress syndrome
Source: J Clin Invest. 2024 Apr 4;134(10):e177896. doi: 10.1172/JCI177896 (PMC11093602; doi:10.1172/JCI177896)
Supplement: Supplemental data set 1 [file jci-134-177896-s011.pdf]

```
*****DATA SETUP*****
drop if subject < 55
drop if subject==.
replace pf24h=50 if pf24h==.
replace oi24h=50 if oi24h==.
gen logvaso = log(vasopressorscore72h+1)
gen persistentards=.
replace persistentards=0 if d7ardsresolved0mild1mod2sev3<2 &
d7ardsresolved0mild1mod2sev3~=.
replace persistentards=1 if d7ardsresolved0mild1mod2sev3>=2 &
d7ardsresolved0mild1mod2sev3~=.
gen pfcat=.
replace pfcat=1 if pfinitial>200
replace pfcat=2 if pfinitial<201 & pfinitial>100
replace pfcat=3 if pfinitial<101
*****pg/mL to ng/mL*****
gen tnfr1ngmlday0= tnfr1pgml/1000
gen tnfr1ngmlday3= tnfr1t13d/1000
gen tnfr1ngmlday7= tnfr1t27d /1000
gen hspngmlday0= hsp70pgml /1000
gen hspngmlday3= hsp70t13d /1000
gen hspngmlday7= hsp70t27d /1000
gen mmpngmlday0= mmp8pgml /1000
gen mmpngmlday3= mmp8t13d /1000
gen mmpngmlday7= mmp8t27d /1000
*****LOG TRANSFORM*****
gen logang0=log( angpt2pgml+1)
gen logang3=log( angptt13d +1)
gen logang7=log( angptt27d +1)
gen lograge0=log( ragepgml+1)
gen lograge3=log( raget13d +1)
gen lograge7=log( raget27d +1)
gen lognucl0=log( nucleosomesnormalized+1)
gen lognucl3=log( nucleosomest1normalizedday3 +1)
gen lognucl7=log( nucleosomest2normalizedday7 +1)
gen logspd0=log( spdngml+1)
gen logspd3=log( spdt13d +1)
gen logspd7=log( spdt27d +1)
gen logp3np0=log( p3npngml+1)
gen logp3np3=log( p3npt13d +1)
gen logp3np7=log( p3npt27d +1)
gen logccl22d0=log( ccl22pgml+1)
gen logccl22d3=log( ccl22t13d +1)
gen logccl22d7=log( ccl22t27d +1)
gen logccl7d0=log( ccl7pgml +1)
gen logccl7d3=log( ccl7t13d +1)
gen logccl7d7=log( ccl7t27d +1)
gen logil6d0=log( il6pgml+1)
gen logil6d3=log( il6t13d +1)
gen logil6d7=log( il6t27d +1)
gen logtnfr10=log( tnfr1pgml+1)
gen logtnfr13=log( tnfr1t13d +1)
gen logtnfr17=log( tnfr1t27d +1)
gen loggzmb0=log( granzymebpgml+1)
```

```
gen loggzmb3=log( granzymebt13d +1)
gen loggzmb7=log( granzymebt27d +1)
gen loghsp0=log( hsp70pgml+1)
gen loghsp3=log( hsp70t13d +1)
gen loghsp7=log( hsp70t27d +1)
gen logil1a0=log( il1apgml+1)
gen logil1a3=log( il1at13d +1)
gen logil1a7=log( il1at27d +1)
gen logil8d0=log( il8pgml+1)
gen logil8d3=log( il8t13d +1)
gen logil8d7=log( il8t27d +1)
gen logmip1a0=log( ccl3mip1apgml+1)
gen logmip1a3=log( ccl3mip1at13d +1)
gen logmip1a7=log( ccl3mip1at27d +1)
gen logmi1b0=log( mip1bpgml+1)
gen logmi1b3=log( mip1bt13d +1)
gen logmi1b7=log( mip1bt27d +1)
gen logmmp0=log( mmp8pgml+1)
gen logmmp3=log( mmp8t13d +1)
gen logmmp7=log( mmp8t27d +1)
gen logcoxi0=log( hcoxicopiesul)
gen logcoxi3=log( hcoxiday3 )
gen logcoxi7=log( hcoxiday7 )
gen lognd0=log( nd1copiesul )
gen lognd3=log( nd1day3 )
gen lognd7=log( nd1day7 )
gen logcoxiv0=log( hcox4copiesul )
gen logcoxiv3=log( hcox4day3 )
gen logcoxiv7=log( hcox4day7 )
*****DEATHS ON DAYS 0, 3, 7*****
set pformat %5.4f
regress  logil1a0 agey i.dxcodedp0s1a2o3 immunosuppresed pfinitial
picudeath
regress  logil6d0 agey i.dxcodedp0s1a2o3 immunosuppresed pfinitial
picudeath
regress  logil8d0 agey i.dxcodedp0s1a2o3 immunosuppresed pfinitial
picudeath
regress  logtnfr10 agey i.dxcodedp0s1a2o3 immunosuppresed pfinitial
picudeath
regress  logmmp0 agey i.dxcodedp0s1a2o3 immunosuppresed pfinitial
picudeath
regress  loggzmb0 agey i.dxcodedp0s1a2o3 immunosuppresed pfinitial
picudeath
regress  logccl7d0 agey i.dxcodedp0s1a2o3 immunosuppresed pfinitial
picudeath
regress  logccl22d0 agey i.dxcodedp0s1a2o3 immunosuppresed pfinitial
picudeath
regress  logmip1a0 agey i.dxcodedp0s1a2o3 immunosuppresed pfinitial
picudeath
regress  logmi1b0 agey i.dxcodedp0s1a2o3 immunosuppresed pfinitial
picudeath
regress  logang0 agey i.dxcodedp0s1a2o3 immunosuppresed pfinitial
picudeath
```

```
regress  logp3np0 agey i.dxcodedp0s1a2o3 immunosuppresed pfinitial
picudeath
regress  lograge0 agey i.dxcodedp0s1a2o3 immunosuppresed pfinitial
picudeath
parmest, list(p) format(p %-8.2g)
regress  logspd0 agey i.dxcodedp0s1a2o3 immunosuppresed pfinitial
picudeath
regress  loghsp0 agey i.dxcodedp0s1a2o3 immunosuppresed pfinitial
picudeath
parmest, list(p) format(p %-8.2g)
regress  lognucl0 agey i.dxcodedp0s1a2o3 immunosuppresed pfinitial
picudeath
parmest, list(p) format(p %-8.2g)
regress  logcoxiv0 agey i.dxcodedp0s1a2o3 immunosuppresed pfinitial
picudeath
parmest, list(p) format(p %-8.2g)
regress  logcoxi0 agey i.dxcodedp0s1a2o3 immunosuppresed pfinitial
picudeath
regress  lognd0 agey i.dxcodedp0s1a2o3 immunosuppresed pfinitial
picudeath
regress  logil1a3 agey i.dxcodedp0s1a2o3 immunosuppresed pfinitial
picudeath
regress  logil6d3 agey i.dxcodedp0s1a2o3 immunosuppresed pfinitial
picudeath
regress  logil8d3 agey i.dxcodedp0s1a2o3 immunosuppresed pfinitial
picudeath
parmest, list(p) format(p %-8.2g)
regress  logtnfr13 agey i.dxcodedp0s1a2o3 immunosuppresed pfinitial
picudeath
parmest, list(p) format(p %-8.2g)
regress  logmmp3 agey i.dxcodedp0s1a2o3 immunosuppresed pfinitial
picudeath
regress  loggzmb3 agey i.dxcodedp0s1a2o3 immunosuppresed pfinitial
picudeath
regress  logccl7d3 agey i.dxcodedp0s1a2o3 immunosuppresed pfinitial
picudeath
regress  logccl22d3 agey i.dxcodedp0s1a2o3 immunosuppresed pfinitial
picudeath
regress  logmip1a3 agey i.dxcodedp0s1a2o3 immunosuppresed pfinitial
picudeath
regress  logmi1b3 agey i.dxcodedp0s1a2o3 immunosuppresed pfinitial
picudeath
regress  logang3 agey i.dxcodedp0s1a2o3 immunosuppresed pfinitial
picudeath
regress  logp3np3 agey i.dxcodedp0s1a2o3 immunosuppresed pfinitial
picudeath
regress  lograge3 agey i.dxcodedp0s1a2o3 immunosuppresed pfinitial
picudeath
parmest, list(p) format(p %-8.2g)
regress  logspd3 agey i.dxcodedp0s1a2o3 immunosuppresed pfinitial
picudeath
regress  loghsp3 agey i.dxcodedp0s1a2o3 immunosuppresed pfinitial
picudeath
parmest, list(p) format(p %-8.2g)
```

```
regress  lognucl3 agey i.dxcodedp0s1a2o3 immunosuppresed pfinitial
picudeath
parmest, list(p) format(p %-8.2g)
regress  logcoxiv3 agey i.dxcodedp0s1a2o3 immunosuppresed pfinitial
picudeath
parmest, list(p) format(p %-8.2g)
regress  logcoxi3 agey i.dxcodedp0s1a2o3 immunosuppresed pfinitial
picudeath
regress  lognd3 agey i.dxcodedp0s1a2o3 immunosuppresed pfinitial
picudeath
regress  logil1a7 agey i.dxcodedp0s1a2o3 immunosuppresed pfinitial
picudeath
regress  logil6d7 agey i.dxcodedp0s1a2o3 immunosuppresed pfinitial
picudeath
regress  logil8d7 agey i.dxcodedp0s1a2o3 immunosuppresed pfinitial
picudeath
parmest, list(p) format(p %-8.2g)
regress  logtnfr17 agey i.dxcodedp0s1a2o3 immunosuppresed pfinitial
picudeath
parmest, list(p) format(p %-8.2g)
regress  logmmp7 agey i.dxcodedp0s1a2o3 immunosuppresed pfinitial
picudeath
regress  loggzmb7 agey i.dxcodedp0s1a2o3 immunosuppresed pfinitial
picudeath
regress  logccl7d7 agey i.dxcodedp0s1a2o3 immunosuppresed pfinitial
picudeath
regress  logccl22d7 agey i.dxcodedp0s1a2o3 immunosuppresed pfinitial
picudeath
regress  logmip1a7 agey i.dxcodedp0s1a2o3 immunosuppresed pfinitial
picudeath
regress  logmi1b7 agey i.dxcodedp0s1a2o3 immunosuppresed pfinitial
picudeath
regress  logang7 agey i.dxcodedp0s1a2o3 immunosuppresed pfinitial
picudeath
regress  logp3np7 agey i.dxcodedp0s1a2o3 immunosuppresed pfinitial
picudeath
regress  lograge7 agey i.dxcodedp0s1a2o3 immunosuppresed pfinitial
picudeath
parmest, list(p) format(p %-8.2g)
regress  logspd7 agey i.dxcodedp0s1a2o3 immunosuppresed pfinitial
picudeath
regress  loghsp7 agey i.dxcodedp0s1a2o3 immunosuppresed pfinitial
picudeath
parmest, list(p) format(p %-8.2g)
regress  lognucl7 agey i.dxcodedp0s1a2o3 immunosuppresed pfinitial
picudeath
regress  logcoxiv7 agey i.dxcodedp0s1a2o3 immunosuppresed pfinitial
picudeath
parmest, list(p) format(p %-8.2g)
regress  logcoxi7 agey i.dxcodedp0s1a2o3 immunosuppresed pfinitial
picudeath
regress  lognd7 agey i.dxcodedp0s1a2o3 immunosuppresed pfinitial
picudeath
*****STANDARDIZE*****
```

```
egen zlogang0=std( logang0 )
egen zlogang3=std( logang3 )
egen zlogang7=std( logang7 )
egen zlograge0=std( lograge0 )
egen zlograge3=std( lograge3 )
egen zlograge7=std( lograge7 )
egen zlognucl0=std( lognucl0 )
egen zlognucl3=std( lognucl3 )
egen zlognucl7=std( lognucl7 )
egen zlogspd0=std( logspd0 )
egen zlogspd3=std( logspd3 )
egen zlogspd7=std( logspd7 )
egen zlogp3np0=std( logp3np0 )
egen zlogp3np3=std( logp3np3 )
egen zlogp3np7=std( logp3np7 )
egen zlogccl22d0=std( logccl22d0 )
egen zlogccl22d3=std( logccl22d3 )
egen zlogccl22d7=std( logccl22d7 )
egen zlogccl7d0=std( logccl7d0 )
egen zlogccl7d3=std( logccl7d3 )
egen zlogccl7d7=std( logccl7d7 )
egen zlogil6d0=std( logil6d0 )
egen zlogil6d3=std( logil6d3 )
egen zlogil6d7=std( logil6d7 )
egen zlogtnfr10=std( logtnfr10 )
egen zlogtnfr13=std( logtnfr13 )
egen zlogtnfr17=std( logtnfr17 )
egen zloggzmb0=std( loggzmb0 )
egen zloggzmb3=std( loggzmb3 )
egen zloggzmb7=std( loggzmb7 )
egen zloghsp0=std( loghsp0 )
egen zloghsp3=std( loghsp3 )
egen zloghsp7=std( loghsp7 )
egen zlogil1a0=std( logil1a0 )
egen zlogil1a3=std( logil1a3 )
egen zlogil1a7=std( logil1a7 )
egen zlogil8d0=std( logil8d0 )
egen zlogil8d3=std( logil8d3 )
egen zlogil8d7=std( logil8d7 )
egen zlogmip1a0=std( logmip1a0 )
egen zlogmip1a3=std( logmip1a3 )
egen zlogmip1a7=std( logmip1a7 )
egen zlogmip1b0=std( logmi1b0 )
egen zlogmip1b3=std( logmi1b3 )
egen zlogmip1b7=std( logmi1b7 )
egen zlogmmp0=std( logmmp0 )
egen zlogmmp3=std( logmmp3 )
egen zlogmmp7=std( logmmp7 )
egen zlogcoxi0=std( logcoxi0 )
egen zlogcoxi3=std( logcoxi3 )
egen zlogcoxi7=std( logcoxi7 )
egen zlognd0=std( lognd0 )
egen zlognd3=std( lognd3 )
egen zlognd7=std( lognd7 )
```

```
egen zlogcoxiv0=std( logcoxiv0 )
egen zlogcoxiv3=std( logcoxiv3 )
egen zlogcoxiv7=std( logcoxiv7 )
*****RESHAPE*****
preserve
reshape long zlogil1a zlogil6d zlogil8d zlogtnfr1 zlogmmp zloggzmb
zlogccl7d zlogccl22d zlogmip1a zlogmip1b zlogang zlogp3np zlograge
zlogspd zloghsp zlognucl zlogcoxiv zlogcoxi zlognd logil1a logil6d
logil8d logtnfr1 logmmp loggzmb logccl7d logccl22d logmip1a logmip1b
logang logp3np lograge logspd loghsp lognucl logcoxiv logcoxi lognd,
i(subject)
*****REGRESS DEATH*****
mixed  zlogil1a agey i.dxcodedp0s1a2o3 immunosuppresed pfinitial
picudeath##c._j || subject:
mixed  zlogil6 agey i.dxcodedp0s1a2o3 immunosuppresed pfinitial
picudeath##c._j || subject:
mixed  zlogil8 agey i.dxcodedp0s1a2o3 immunosuppresed pfinitial
picudeath##c._j || subject:
mixed  zlogtnfr1 agey i.dxcodedp0s1a2o3 immunosuppresed pfinitial
picudeath##c._j || subject:
mixed  zlogmmp agey i.dxcodedp0s1a2o3 immunosuppresed pfinitial
picudeath##c._j || subject:
mixed  zloggzmb agey i.dxcodedp0s1a2o3 immunosuppresed pfinitial
picudeath##c._j || subject:
mixed  zlogccl7d agey i.dxcodedp0s1a2o3 immunosuppresed pfinitial
picudeath##c._j || subject:
mixed  zlogccl22d agey i.dxcodedp0s1a2o3 immunosuppresed pfinitial
picudeath##c._j || subject:
mixed  zlogmip1a agey i.dxcodedp0s1a2o3 immunosuppresed pfinitial
picudeath##c._j || subject:
mixed  zlogmip1b agey i.dxcodedp0s1a2o3 immunosuppresed pfinitial
picudeath##c._j || subject:
mixed  zlogang agey i.dxcodedp0s1a2o3 immunosuppresed pfinitial
picudeath##c._j || subject:
mixed  zlogp3np agey i.dxcodedp0s1a2o3 immunosuppresed pfinitial
picudeath##c._j || subject:
mixed  zlograge agey i.dxcodedp0s1a2o3 immunosuppresed pfinitial
picudeath##c._j || subject:
mixed  zlogspd agey i.dxcodedp0s1a2o3 immunosuppresed pfinitial
picudeath##c._j || subject:
mixed  zloghsp agey i.dxcodedp0s1a2o3 immunosuppresed pfinitial
picudeath##c._j || subject:
mixed  zlognucl agey i.dxcodedp0s1a2o3 immunosuppresed pfinitial
picudeath##c._j || subject:
mixed  zlogcoxiv agey i.dxcodedp0s1a2o3 immunosuppresed pfinitial
picudeath##c._j || subject:
mixed  zlogcoxi agey i.dxcodedp0s1a2o3 immunosuppresed pfinitial
picudeath##c._j || subject:
mixed  zlognd agey i.dxcodedp0s1a2o3 immunosuppresed pfinitial
picudeath##c._j || subject:
*****REGRESS DIRECT*****
mixed  zlogil1a agey  immunosuppresed pfinitial direct0##c._j || subject:
mixed  zlogil6 agey  immunosuppresed pfinitial direct0##c._j || subject:
mixed  zlogil8 agey  immunosuppresed pfinitial direct0##c._j || subject:
```

```
mixed  zlogtnfr1 agey  immunosuppresed pfinitial direct0##c._j ||
subject:
mixed  zlogmmp agey  immunosuppresed pfinitial direct0##c._j || subject:
mixed  zloggzmb agey  immunosuppresed pfinitial direct0##c._j || subject:
mixed  zlogccl7d agey  immunosuppresed pfinitial direct0##c._j ||
subject:
mixed  zlogccl22d agey  immunosuppresed pfinitial direct0##c._j ||
subject:
mixed  zlogmip1a agey  immunosuppresed pfinitial direct0##c._j ||
subject:
mixed  zlogmip1b agey  immunosuppresed pfinitial direct0##c._j ||
subject:
mixed  zlogang agey  immunosuppresed pfinitial direct0##c._j || subject:
mixed  zlogp3np agey  immunosuppresed pfinitial direct0##c._j || subject:
mixed  zlograge agey  immunosuppresed pfinitial direct0##c._j || subject:
mixed  zlogspd agey  immunosuppresed pfinitial direct0##c._j || subject:
mixed  zloghsp agey  immunosuppresed pfinitial direct0##c._j || subject:
mixed  zlognucl agey  immunosuppresed pfinitial direct0##c._j || subject:
mixed  zlogcoxiv agey  immunosuppresed pfinitial direct0##c._j ||
subject:
mixed  zlogcoxi agey  immunosuppresed pfinitial direct0##c._j || subject:
mixed  zlognd agey  immunosuppresed pfinitial direct0##c._j || subject:
*****REGRESS PERSISTENT ARDS*****
mixed  zlogil1a agey i.dxcodedp0s1a2o3 immunosuppresed pfinitial
persistentards##c._j || subject:
mixed  zlogil6 agey i.dxcodedp0s1a2o3 immunosuppresed pfinitial
persistentards##c._j || subject:
mixed  zlogil8 agey i.dxcodedp0s1a2o3 immunosuppresed pfinitial
persistentards##c._j || subject:
mixed  zlogtnfr1 agey i.dxcodedp0s1a2o3 immunosuppresed pfinitial
persistentards##c._j || subject:
mixed  zlogmmp agey i.dxcodedp0s1a2o3 immunosuppresed pfinitial
persistentards##c._j || subject:
mixed  zloggzmb agey i.dxcodedp0s1a2o3 immunosuppresed pfinitial
persistentards##c._j || subject:
mixed  zlogccl7d agey i.dxcodedp0s1a2o3 immunosuppresed pfinitial
persistentards##c._j || subject:
mixed  zlogccl22d agey i.dxcodedp0s1a2o3 immunosuppresed pfinitial
persistentards##c._j || subject:
mixed  zlogmip1a agey i.dxcodedp0s1a2o3 immunosuppresed pfinitial
persistentards##c._j || subject:
mixed  zlogmip1b agey i.dxcodedp0s1a2o3 immunosuppresed pfinitial
persistentards##c._j || subject:
mixed  zlogang agey i.dxcodedp0s1a2o3 immunosuppresed pfinitial
persistentards##c._j || subject:
mixed  zlogp3np agey i.dxcodedp0s1a2o3 immunosuppresed pfinitial
persistentards##c._j || subject:
mixed  zlograge agey i.dxcodedp0s1a2o3 immunosuppresed pfinitial
persistentards##c._j || subject:
mixed  zlogspd agey i.dxcodedp0s1a2o3 immunosuppresed pfinitial
persistentards##c._j || subject:
mixed  zloghsp agey i.dxcodedp0s1a2o3 immunosuppresed pfinitial
persistentards##c._j || subject:
```

```
mixed  zlognucl agey i.dxcodedp0s1a2o3 immunosuppresed pfinitial
persistentards##c._j || subject:
mixed  zlogcoxiv agey i.dxcodedp0s1a2o3 immunosuppresed pfinitial
persistentards##c._j || subject:
mixed  zlogcoxi agey i.dxcodedp0s1a2o3 immunosuppresed pfinitial
persistentards##c._j || subject:
mixed  zlognd agey i.dxcodedp0s1a2o3 immunosuppresed pfinitial
persistentards##c._j || subject:
*****REGRESS MODS*****
mixed  zlogil1a agey i.dxcodedp0s1a2o3 immunosuppresed pfinitial
modsd7##c._j || subject:
mixed  zlogil6 agey i.dxcodedp0s1a2o3 immunosuppresed pfinitial
modsd7##c._j || subject:
mixed  zlogil8 agey i.dxcodedp0s1a2o3 immunosuppresed pfinitial
modsd7##c._j || subject:
mixed  zlogtnfr1 agey i.dxcodedp0s1a2o3 immunosuppresed pfinitial
modsd7##c._j || subject:
mixed  zlogmmp agey i.dxcodedp0s1a2o3 immunosuppresed pfinitial
modsd7##c._j || subject:
mixed  zloggzmb agey i.dxcodedp0s1a2o3 immunosuppresed pfinitial
modsd7##c._j || subject:
mixed  zlogccl7d agey i.dxcodedp0s1a2o3 immunosuppresed pfinitial
modsd7##c._j || subject:
mixed  zlogccl22d agey i.dxcodedp0s1a2o3 immunosuppresed pfinitial
modsd7##c._j || subject:
mixed  zlogmip1a agey i.dxcodedp0s1a2o3 immunosuppresed pfinitial
modsd7##c._j || subject:
mixed  zlogmip1b agey i.dxcodedp0s1a2o3 immunosuppresed pfinitial
modsd7##c._j || subject:
mixed  zlogang agey i.dxcodedp0s1a2o3 immunosuppresed pfinitial
modsd7##c._j || subject:
mixed  zlogp3np agey i.dxcodedp0s1a2o3 immunosuppresed pfinitial
modsd7##c._j || subject:
mixed  zlograge agey i.dxcodedp0s1a2o3 immunosuppresed pfinitial
modsd7##c._j || subject:
mixed  zlogspd agey i.dxcodedp0s1a2o3 immunosuppresed pfinitial
modsd7##c._j || subject:
mixed  zloghsp agey i.dxcodedp0s1a2o3 immunosuppresed pfinitial
modsd7##c._j || subject:
mixed  zlognucl agey i.dxcodedp0s1a2o3 immunosuppresed pfinitial
modsd7##c._j || subject:
mixed  zlogcoxiv agey i.dxcodedp0s1a2o3 immunosuppresed pfinitial
modsd7##c._j || subject:
mixed  zlogcoxi agey i.dxcodedp0s1a2o3 immunosuppresed pfinitial
modsd7##c._j || subject:
mixed  zlognd agey i.dxcodedp0s1a2o3 immunosuppresed pfinitial
modsd7##c._j || subject:
*****REGRESS ICC*****
mixed  zlogil1a agey i.dxcodedp0s1a2o3  pfinitial immunosuppresed##c._j
|| subject:
mixed  zlogil6 agey i.dxcodedp0s1a2o3 pfinitial immunosuppresed##c._j ||
subject:
mixed  zlogtnfr1 agey i.dxcodedp0s1a2o3 pfinitial immunosuppresed##c._j
|| subject:
```

```
mixed  zlogmmp agey i.dxcodedp0s1a2o3 pfinitial immunosuppresed##c._j ||
subject:
mixed  zloggzmb agey i.dxcodedp0s1a2o3 pfinitial immunosuppresed##c._j ||
subject:
mixed  zlogil8 agey i.dxcodedp0s1a2o3 pfinitial immunosuppresed##c._j ||
subject:
mixed  zlogccl7d agey i.dxcodedp0s1a2o3 pfinitial immunosuppresed##c._j
|| subject:
mixed  zlogccl22d agey i.dxcodedp0s1a2o3 pfinitial immunosuppresed##c._j
|| subject:
mixed  zlogmip1a agey i.dxcodedp0s1a2o3 pfinitial immunosuppresed##c._j
|| subject:
mixed  zlogmip1b agey i.dxcodedp0s1a2o3 pfinitial immunosuppresed##c._j
|| subject:
mixed  zlogang agey i.dxcodedp0s1a2o3 pfinitial immunosuppresed##c._j ||
subject:
mixed  zlogp3np agey i.dxcodedp0s1a2o3 pfinitial immunosuppresed##c._j ||
subject:
mixed  zlograge agey i.dxcodedp0s1a2o3 pfinitial immunosuppresed##c._j ||
subject:
mixed  zlogspd agey i.dxcodedp0s1a2o3 pfinitial immunosuppresed##c._j ||
subject:
mixed  zloghsp agey i.dxcodedp0s1a2o3 pfinitial immunosuppresed##c._j ||
subject:
mixed  zlognucl agey i.dxcodedp0s1a2o3 pfinitial immunosuppresed##c._j ||
subject:
mixed  zlogcoxiv agey i.dxcodedp0s1a2o3 pfinitial immunosuppresed##c._j
|| subject:
mixed  zlogcoxi agey i.dxcodedp0s1a2o3 pfinitial immunosuppresed##c._j ||
subject:
mixed  zlognd agey i.dxcodedp0s1a2o3 pfinitial immunosuppresed##c._j ||
subject:
*****REGRESS STEROIDS*****
mixed  zlogil1a agey i.dxcodedp0s1a2o3 immunosuppresed pfinitial
steroidsyn##c._j || subject:
mixed  zlogil6 agey i.dxcodedp0s1a2o3 immunosuppresed pfinitial
steroidsyn##c._j || subject:
mixed  zlogtnfr1 agey i.dxcodedp0s1a2o3 immunosuppresed pfinitial
steroidsyn##c._j || subject:
mixed  zlogmmp agey i.dxcodedp0s1a2o3 immunosuppresed pfinitial
steroidsyn##c._j || subject:
mixed  zloggzmb agey i.dxcodedp0s1a2o3 immunosuppresed pfinitial
steroidsyn##c._j || subject:
mixed  zlogil8 agey i.dxcodedp0s1a2o3 immunosuppresed pfinitial
steroidsyn##c._j || subject:
mixed  zlogccl7d agey i.dxcodedp0s1a2o3 immunosuppresed pfinitial
steroidsyn##c._j || subject:
mixed  zlogccl22d agey i.dxcodedp0s1a2o3 immunosuppresed pfinitial
steroidsyn##c._j || subject:
mixed  zlogmip1a agey i.dxcodedp0s1a2o3 immunosuppresed pfinitial
steroidsyn##c._j || subject:
mixed  zlogmip1b agey i.dxcodedp0s1a2o3 immunosuppresed pfinitial
steroidsyn##c._j || subject:
```

```
mixed  zlogang agey i.dxcodedp0s1a2o3 immunosuppresed pfinitial
steroidsyn##c._j || subject:
mixed  zlogp3np agey i.dxcodedp0s1a2o3 immunosuppresed pfinitial
steroidsyn##c._j || subject:
mixed  zlograge agey i.dxcodedp0s1a2o3 immunosuppresed pfinitial
steroidsyn##c._j || subject:
mixed  zlogspd agey i.dxcodedp0s1a2o3 immunosuppresed pfinitial
steroidsyn##c._j || subject:
mixed  zloghsp agey i.dxcodedp0s1a2o3 immunosuppresed pfinitial
steroidsyn##c._j || subject:
mixed  zlognucl agey i.dxcodedp0s1a2o3 immunosuppresed pfinitial
steroidsyn##c._j || subject:
mixed  zlogcoxiv agey i.dxcodedp0s1a2o3 immunosuppresed pfinitial
steroidsyn##c._j || subject:
mixed  zlogcoxi agey i.dxcodedp0s1a2o3 immunosuppresed pfinitial
steroidsyn##c._j || subject:
mixed  zlognd agey i.dxcodedp0s1a2o3 immunosuppresed pfinitial
steroidsyn##c._j || subject:
*****REGRESS ICC MORTALITY*****
mixed  zlogil1a agey i.dxcodedp0s1a2o3  pfinitial picudeath##c._j if
immunosuppresed==0 || subject:
mixed  zlogil6 agey i.dxcodedp0s1a2o3  pfinitial picudeath##c._j if
immunosuppresed==0 || subject:
mixed  zlogtnfr1 agey i.dxcodedp0s1a2o3  pfinitial picudeath##c._j if
immunosuppresed==0 || subject:
mixed  zlogmmp agey i.dxcodedp0s1a2o3  pfinitial picudeath##c._j if
immunosuppresed==0 || subject:
mixed  zloggzmb agey i.dxcodedp0s1a2o3  pfinitial picudeath##c._j if
immunosuppresed==0 || subject:
mixed  zlogil8 agey i.dxcodedp0s1a2o3  pfinitial picudeath##c._j if
immunosuppresed==0 || subject:
mixed  zlogccl7d agey i.dxcodedp0s1a2o3  pfinitial picudeath##c._j if
immunosuppresed==0 || subject:
mixed  zlogccl22d agey i.dxcodedp0s1a2o3  pfinitial picudeath##c._j if
immunosuppresed==0 || subject:
mixed  zlogmip1a agey i.dxcodedp0s1a2o3  pfinitial picudeath##c._j if
immunosuppresed==0 || subject:
mixed  zlogmip1b agey i.dxcodedp0s1a2o3  pfinitial picudeath##c._j if
immunosuppresed==0 || subject:
mixed  zlogang agey i.dxcodedp0s1a2o3  pfinitial picudeath##c._j if
immunosuppresed==0 || subject:
mixed  zlogp3np agey i.dxcodedp0s1a2o3  pfinitial picudeath##c._j if
immunosuppresed==0 || subject:
mixed  zlograge agey i.dxcodedp0s1a2o3  pfinitial picudeath##c._j if
immunosuppresed==0 || subject:
mixed  zlogspd agey i.dxcodedp0s1a2o3  pfinitial picudeath##c._j if
immunosuppresed==0 || subject:
mixed  zloghsp agey i.dxcodedp0s1a2o3  pfinitial picudeath##c._j if
immunosuppresed==0 || subject:
mixed  zlognucl agey i.dxcodedp0s1a2o3  pfinitial picudeath##c._j if
immunosuppresed==0 || subject:
mixed  zlogcoxiv agey i.dxcodedp0s1a2o3  pfinitial picudeath##c._j if
immunosuppresed==0 || subject:
```

```
mixed  zlogcoxi agey i.dxcodedp0s1a2o3  pfinitial picudeath##c._j if
immunosuppresed==0 || subject:
mixed  zlognd agey i.dxcodedp0s1a2o3  pfinitial picudeath##c._j if
immunosuppresed==0 || subject:
mixed  zlogil1a agey i.dxcodedp0s1a2o3  pfinitial picudeath##c._j if
immunosuppresed==1 || subject:
mixed  zlogil6 agey i.dxcodedp0s1a2o3  pfinitial picudeath##c._j if
immunosuppresed==1 || subject:
mixed  zlogtnfr1 agey i.dxcodedp0s1a2o3  pfinitial picudeath##c._j if
immunosuppresed==1 || subject:
mixed  zlogmmp agey i.dxcodedp0s1a2o3  pfinitial picudeath##c._j if
immunosuppresed==1 || subject:
mixed  zloggzmb agey i.dxcodedp0s1a2o3  pfinitial picudeath##c._j if
immunosuppresed==1 || subject:
mixed  zlogil8 agey i.dxcodedp0s1a2o3  pfinitial picudeath##c._j if
immunosuppresed==1 || subject:
mixed  zlogccl7d agey i.dxcodedp0s1a2o3  pfinitial picudeath##c._j if
immunosuppresed==1 || subject:
mixed  zlogccl22d agey i.dxcodedp0s1a2o3  pfinitial picudeath##c._j if
immunosuppresed==1 || subject:
mixed  zlogmip1a agey i.dxcodedp0s1a2o3  pfinitial picudeath##c._j if
immunosuppresed==1 || subject:
mixed  zlogmip1b agey i.dxcodedp0s1a2o3  pfinitial picudeath##c._j if
immunosuppresed==1 || subject:
mixed  zlogang agey i.dxcodedp0s1a2o3  pfinitial picudeath##c._j if
immunosuppresed==1 || subject:
mixed  zlogp3np agey i.dxcodedp0s1a2o3  pfinitial picudeath##c._j if
immunosuppresed==1 || subject:
mixed  zlograge agey i.dxcodedp0s1a2o3  pfinitial picudeath##c._j if
immunosuppresed==1 || subject:
mixed  zlogspd agey i.dxcodedp0s1a2o3  pfinitial picudeath##c._j if
immunosuppresed==1 || subject:
mixed  zloghsp agey i.dxcodedp0s1a2o3  pfinitial picudeath##c._j if
immunosuppresed==1 || subject:
mixed  zlognucl agey i.dxcodedp0s1a2o3  pfinitial picudeath##c._j if
immunosuppresed==1 || subject:
mixed  zlogcoxiv agey i.dxcodedp0s1a2o3  pfinitial picudeath##c._j if
immunosuppresed==1 || subject:
mixed  zlogcoxi agey i.dxcodedp0s1a2o3  pfinitial picudeath##c._j if
immunosuppresed==1 || subject:
mixed  zlognd agey i.dxcodedp0s1a2o3  pfinitial picudeath##c._j if
immunosuppresed==1 || subject:
*****REGRESS STEROID MORTALITY*****
mixed  zlogil1a agey i.dxcodedp0s1a2o3 immunosuppresed pfinitial
picudeath##c._j if steroidsyn==0 || subject:
mixed  zlogil6 agey i.dxcodedp0s1a2o3 immunosuppresed pfinitial
picudeath##c._j if steroidsyn==0 || subject:
mixed  zlogtnfr1 agey i.dxcodedp0s1a2o3 immunosuppresed pfinitial
picudeath##c._j if steroidsyn==0 || subject:
mixed  zlogmmp agey i.dxcodedp0s1a2o3 immunosuppresed pfinitial
picudeath##c._j if steroidsyn==0 || subject:
mixed  zloggzmb agey i.dxcodedp0s1a2o3 immunosuppresed pfinitial
picudeath##c._j if steroidsyn==0 || subject:
```

```
mixed  zlogil8 agey i.dxcodedp0s1a2o3 immunosuppresed pfinitial
picudeath##c._j if steroidsyn==0 || subject:
mixed  zlogccl7d agey i.dxcodedp0s1a2o3 immunosuppresed pfinitial
picudeath##c._j if steroidsyn==0 || subject:
mixed  zlogccl22d agey i.dxcodedp0s1a2o3 immunosuppresed pfinitial
picudeath##c._j if steroidsyn==0 || subject:
mixed  zlogmip1a agey i.dxcodedp0s1a2o3 immunosuppresed pfinitial
picudeath##c._j if steroidsyn==0 || subject:
mixed  zlogmip1b agey i.dxcodedp0s1a2o3 immunosuppresed pfinitial
picudeath##c._j if steroidsyn==0 || subject:
mixed  zlogang agey i.dxcodedp0s1a2o3 immunosuppresed pfinitial
picudeath##c._j if steroidsyn==0 || subject:
mixed  zlogp3np agey i.dxcodedp0s1a2o3 immunosuppresed pfinitial
picudeath##c._j if steroidsyn==0 || subject:
mixed  zlograge agey i.dxcodedp0s1a2o3 immunosuppresed pfinitial
picudeath##c._j if steroidsyn==0 || subject:
mixed  zlogspd agey i.dxcodedp0s1a2o3 immunosuppresed pfinitial
picudeath##c._j if steroidsyn==0 || subject:
mixed  zloghsp agey i.dxcodedp0s1a2o3 immunosuppresed pfinitial
picudeath##c._j if steroidsyn==0 || subject:
mixed  zlognucl agey i.dxcodedp0s1a2o3 immunosuppresed pfinitial
picudeath##c._j if steroidsyn==0 || subject:
mixed  zlogcoxiv agey i.dxcodedp0s1a2o3 immunosuppresed pfinitial
picudeath##c._j if steroidsyn==0 || subject:
mixed  zlogcoxi agey i.dxcodedp0s1a2o3 immunosuppresed pfinitial
picudeath##c._j if steroidsyn==0 || subject:
mixed  zlognd agey i.dxcodedp0s1a2o3 immunosuppresed pfinitial
picudeath##c._j if steroidsyn==0 || subject:
mixed  zlogil1a agey i.dxcodedp0s1a2o3 immunosuppresed pfinitial
picudeath##c._j if steroidsyn==1 || subject:
mixed  zlogil6 agey i.dxcodedp0s1a2o3 immunosuppresed pfinitial
picudeath##c._j if steroidsyn==1 || subject:
mixed  zlogtnfr1 agey i.dxcodedp0s1a2o3 immunosuppresed pfinitial
picudeath##c._j if steroidsyn==1 || subject:
mixed  zlogmmp agey i.dxcodedp0s1a2o3 immunosuppresed pfinitial
picudeath##c._j if steroidsyn==1 || subject:
mixed  zloggzmb agey i.dxcodedp0s1a2o3 immunosuppresed pfinitial
picudeath##c._j if steroidsyn==1 || subject:
mixed  zlogil8 agey i.dxcodedp0s1a2o3 immunosuppresed pfinitial
picudeath##c._j if steroidsyn==1 || subject:
mixed  zlogccl7d agey i.dxcodedp0s1a2o3 immunosuppresed pfinitial
picudeath##c._j if steroidsyn==1 || subject:
mixed  zlogccl22d agey i.dxcodedp0s1a2o3 immunosuppresed pfinitial
picudeath##c._j if steroidsyn==1 || subject:
mixed  zlogmip1a agey i.dxcodedp0s1a2o3 immunosuppresed pfinitial
picudeath##c._j if steroidsyn==1 || subject:
mixed  zlogmip1b agey i.dxcodedp0s1a2o3 immunosuppresed pfinitial
picudeath##c._j if steroidsyn==1 || subject:
mixed  zlogang agey i.dxcodedp0s1a2o3 immunosuppresed pfinitial
picudeath##c._j if steroidsyn==1 || subject:
mixed  zlogp3np agey i.dxcodedp0s1a2o3 immunosuppresed pfinitial
picudeath##c._j if steroidsyn==1 || subject:
mixed  zlograge agey i.dxcodedp0s1a2o3 immunosuppresed pfinitial
picudeath##c._j if steroidsyn==1 || subject:
```

```
mixed  zlogspd agey i.dxcodedp0s1a2o3 immunosuppresed pfinitial
picudeath##c._j if steroidsyn==1 || subject:
mixed  zloghsp agey i.dxcodedp0s1a2o3 immunosuppresed pfinitial
picudeath##c._j if steroidsyn==1 || subject:
mixed  zlognucl agey i.dxcodedp0s1a2o3 immunosuppresed pfinitial
picudeath##c._j if steroidsyn==1 || subject:
mixed  zlogcoxiv agey i.dxcodedp0s1a2o3 immunosuppresed pfinitial
picudeath##c._j if steroidsyn==1 || subject:
mixed  zlogcoxi agey i.dxcodedp0s1a2o3 immunosuppresed pfinitial
picudeath##c._j if steroidsyn==1 || subject:
mixed  zlognd agey i.dxcodedp0s1a2o3 immunosuppresed pfinitial
picudeath##c._j if steroidsyn==1 || subject:
```
